# Restoring mitochondrial cardiolipin homeostasis reduces cell death and promotes recovery after spinal cord injury

**DOI:** 10.1038/s41419-022-05369-5

**Published:** 2022-12-20

**Authors:** Nai-Kui Liu, Ling-Xiao Deng, Miao Wang, Qing-Bo Lu, Chunyan Wang, Xiangbing Wu, Wei Wu, Ying Wang, Wenrui Qu, Qi Han, Yongzhi Xia, Baylen Ravenscraft, Jin-Lian Li, Si-Wei You, Peter Wipf, Xianlin Han, Xiao-Ming Xu

**Affiliations:** 1 Spinal Cord and Brain Injury Research Group, Stark Neurosciences Research Institute, Department of Neurological Surgery, Indiana University School of Medicine, Indianapolis, IN 46202 USA; 2 Frontage Laboratories, Exton, PA 19341 USA; 3 Department of Medicine, Washington University School of Medicine, St. Louis, MO 63110 USA; 4 Department of Anatomy and K.K. Leung Brain Research Centre, Preclinical School of Medicine, The Fourth Military Medical University, Xi’an, 710032 P. R. China; 5 Institute of Neuroscience, The Fourth Military Medical University, Xi’an, P. R. China; 6 Department of Chemistry, University of Pittsburgh, Pittsburgh, PA 15260 USA; 7 Department of Medicine, University of Texas Health Science Center at San Antonio, San Antonio, TX 78229 USA

**Keywords:** Spinal cord diseases, Cell death in the nervous system

## Abstract

Alterations in phospholipids have long been associated with spinal cord injury (SCI). However, their specific roles and signaling cascades in mediating cell death and tissue repair remain unclear. Here we investigated whether alterations of cardiolipin (CL), a family of mitochondrion-specific phospholipids, play a crucial role in mitochondrial dysfunction and neuronal death following SCI. Lipidomic analysis was used to determine the profile of CL alteration in the adult rat spinal cord following a moderate contusive SCI at the 10th thoracic (T10) level. Cellular, molecular, and genetic assessments were performed to determine whether CL alterations mediate mitochondrial dysfunction and neuronal death after SCI, and, if so, whether reversing CL alteration leads to neuroprotection after SCI. Using lipidomic analysis, we uncovered CL alterations at an early stage of SCI. Over 50 distinct CL species were identified, of which 50% showed significantly decreased abundance after SCI. The decreased CL species contained mainly polyunsaturated fatty acids that are highly susceptible to peroxidation. In parallel, 4-HNE, a lipid peroxidation marker, significantly increased after SCI. We found that mitochondrial oxidative stress not only induced CL oxidation, but also resulted in CL loss by activating cPLA_2_ to hydrolyze CL. CL alterations induced mitochondrial dysfunction and neuronal death. Remarkably, pharmacologic inhibition of CL alterations with XJB-5-131, a novel mitochondria-targeted electron and reactive oxygen species scavenger, reduced cell death, tissue damage and ameliorated motor deficits after SCI in adult rats. These findings suggest that CL alteration could be a novel mechanism that mediates injury-induced neuronal death, and a potential therapeutic target for ameliorating secondary SCI.

## Introduction

Traumatic spinal cord injury (SCI) leads to neurological deficits below the level of injury. In the United States alone there were approximately 282,000 people living with SCI in 2016, and an additional 17,000 new SCI cases occur every year [1]. To date, there has been no effective treatment available for SCI patients [2].

Phospholipids are the main components of a neural cell bi-layer membrane. They not only constitute the backbone of a neural membrane, but also provide the membrane with suitable physical properties, fluidity, and ion permeability, which are required for the proper function of integral membrane proteins, receptors, and ion channels [3–5]. Increasing evidence points to the roles of phospholipids as signaling molecules, as participants and coordinators of responses to physiological regulation, and as danger signals [6–8]. Although phospholipid alterations have been implicated in SCI [9–12], their profiles and specific roles in mediating damage remain elusive.

Cardiolipin (CL) is a class of structurally unique dimeric phospholipids that is exclusively present in the inner mitochondrial membrane (IMM) where it is required for optimal mitochondrial function [13, 14]. In addition to its role in maintaining membrane potential and architecture, CL is known to provide essential structural and functional support to several proteins involved in mitochondrial bioenergetics [13–15]. Recently, CL is emerging as an important player in the control of the mitochondrial phase of apoptosis [13, 14, 16]. CL alteration (peroxidation and/or loss of CL content) has been implicated in mitochondrial dysfunction and cell death in multiple tissues in a variety of pathological conditions including ischemia, hypothyroidism, aging, heart failure, and traumatic brain injury [13, 17–19]. However, limited information is available concerning its role and underlying mechanism following a trauma, such as a SCI. In this study, we employed lipidomics to investigate CL alterations following SCI, and explored the molecular mechanisms of its alterations. We used both in vitro and in vivo experiments to determine whether CL alteration would contribute to the pathogenesis of cell death and functional impairments following SCI.

## Results

### An increase in biomarkers of apoptosis indicates cardiolipin alteration after SCI

**Fig. 1:**
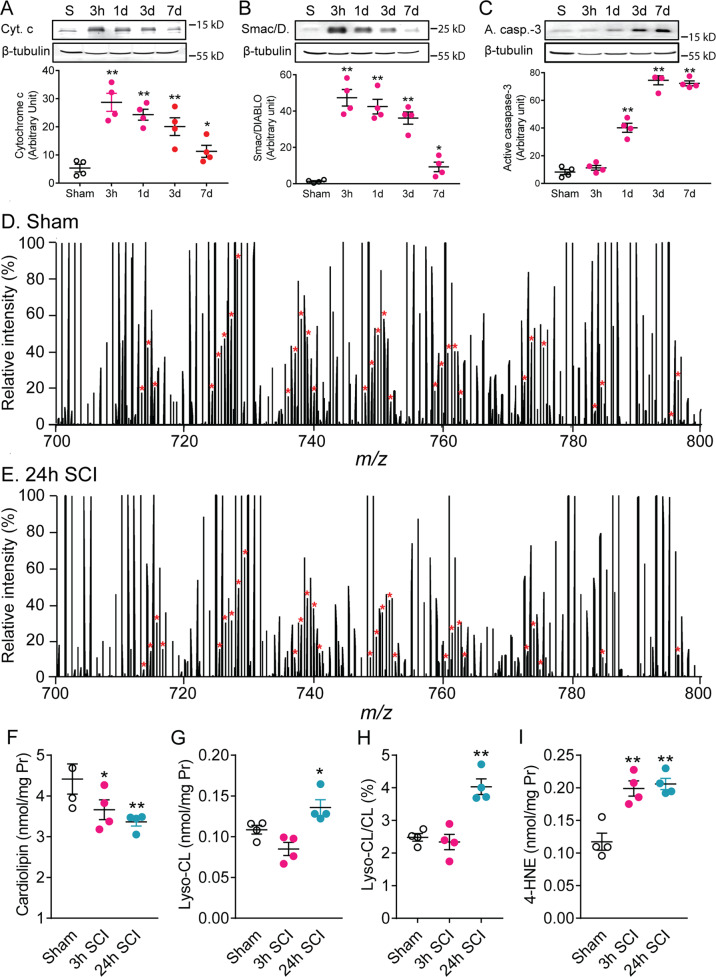
Cardiolipin (CL) alteration in the injured spinal cord following spinal cord injury (SCI). **A**–**C** Biomarkers of apoptosis following SCI. Time course of cytochrome c release (**A**), Smac/DIABLO release (**B**), and active caspase-3 expression (**C**) following SCI. *P < 0.05, **P < 0.01 vs sham (One-way ANOVA, Dunnett post-test, *n* = 4 rats/group). Data represent mean ± s.e.m. Cyt. c cytochrome c, Smac/D. Smac/DIABLO, A. casp.-3 active casepase-3. **D**–**I** Lipidomic analysis after SCI. Lipid extracts of spinal cord from sham, 3 h, and 24 h SCI rats were prepared by using the methyl-tert-butyl ether (MTBE) extraction. **D**, **E** Expanded negative-ion ESI mass spectra of rat spinal cord lipid extracts obtained using a Q Exactive™ Hybrid Quadrupole-Orbitrap mass spectrometer. The asterisks indicate the identified CL plus-one isotopologues, which were characteristic of the doubly charged CL molecular species and were used to quantify individual CL molecular species. Each spectrum is displayed after being normalized to the intensity of internal standard 1’, 3’-bis [1, 2-dimyristoyl-sn-glycero-3-phospho]-*sn*-glycerol (tetra14:0 CL, [CL-2H + 1]^2-^ = 619.91791) ion. The *x*-axis is mass-to-charge ratio (*m/z*). A significant decrease in CL was detected at 3 and 24 h post-injury (**D**–**F**) while a corresponding increase in lyso-CL and ratio of lyso-CL/CL was observed at 24 h post-injury (**G**, **H**). 4-HNE, a marker of lipid peroxidation, was significantly increased at 3 and 24 h after SCI (**I**). Data represent mean ± s.e.m. *P < 0.05, **P < 0.01 (One-way ANOVA, Tukey’s multiple comparisons test, *n* = 4 rats/groups). CL cardiolipin, Lyso-CL lyso-cardiolipin, 4-HNE 4-hydroxy Nonenal.

CL is a class of mitochondrial phospholipids which are known to be intimately linked to the mitochondrial bioenergetic machinery [13, 14]. In recent years, CL has been increasingly recognized as playing a key role in mitochondria-mediated apoptosis [13, 14, 16]. CL loss causes the release of cytochrome c and Smac/DIABLO, as well as an increase in caspase-3 activity during the induction of apoptosis [16, 20, 21]. Simultaneous measurements of cytochrome c release, Smac/DIABLO release, and caspase-3 activity can be used to assess the extent of cardiolipin loss. Western blot analysis revealed that both releases of cytochrome c (Fig. 1A) and Smac/DIABLO (Fig. 1B) markedly increased and peaked at 3 h post-SCI compared to the control. Although both releases declined after 1 day post-injury, they remained at significantly elevated levels up to 7 days post-injury (*p* < 0.05) (Fig. 1A, B). Expression of active caspase-3 was also markedly increased at 1 day post-injury, and reached its peak at 3 and 7 days post-injury (Fig. 1C, Supplementary Fig. 1). These findings suggest that SCI may induce CL alteration.

### Lipidomic analysis shows cardiolipin alterations in the spinal cord after SCI

To further determine CL alteration after SCI, molecular species of CL were characterized by electrospray ionization mass spectrometry (ESI-MS) using the negative-ion mode as previously described [22]_._ Representative ESI-MS spectra of lipids in the spinal cord with and without injury are presented in Fig. 1D, E. Our lipidomic analysis showed that CL was significantly reduced (Fig. 1D–F) and 4-hydroxy nonenal (4-HNE), a lipid peroxidation marker, significantly increased at 3 and 24 h after SCI in a rat contusive SCI (Fig. 1I). Furthermore, lyso-CL (Fig. 1G) and the ratio of lyso-CL/CL (Fig. 1H) increased at 24 h after SCI. These results suggest that CL may undergo peroxidation at 3 h, and both peroxidation and loss at 24 h after SCI.

### Lipidomic analysis identifies changes of molecular species and acyl composition of cardiolipin after SCI

**Fig. 2:**
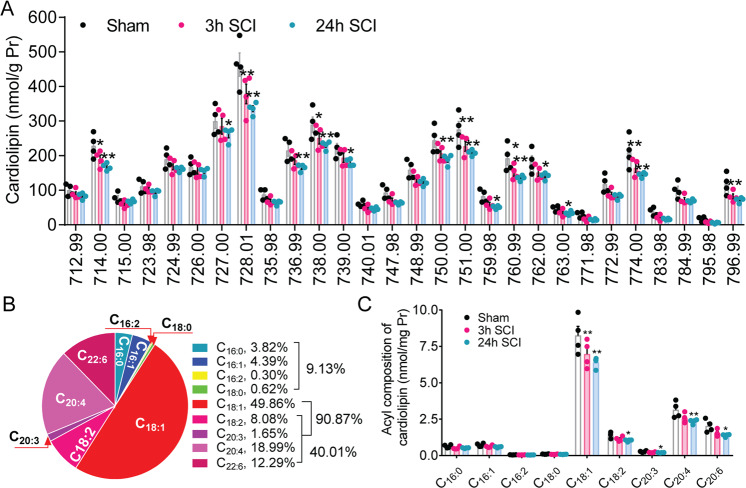
Quantification of molecular species and acyl composition of cardiolipin (CL) in the injured spinal cord at 3 and 24 h after SCI. **A** Quantification of CL molecular species at 3 and 24 h after SCI. Over 50 distinct CL molecular species were readily identified. Of them, 50% of species of CL were significantly reduced after SCI. *P < 0.05, **P < 0.01 vs. sham (Two-way ANOVA, Tukey’s multiple comparisons test, *n* = 4 rats/groups). Data represent mean ± s.e.m. The *x*-axis is mass-to-charge ratio (m/z). **B**, **C** Quantification of acyl composition in CL species at 3 and 24 h after SCI. 18:1 fatty acid was predominantly composed of CL and was 49.86% of CL compositions. The following enrichment of acyl was 20:4 (AA, 18.99%), 22:6 (DHA, 12.29%) and 18:2 (LA, 8.08%). After SCI, all decreased CL acyls were unsaturated fatty acids; of them, 50% were poly-unsaturated fatty acids. *P < 0.05, **P < 0.01 vs. sham (Two-way ANOVA, Tukey’s multiple comparisons test, *n* = 4 rats/groups). Data represent mean ± s.e.m.

The profile of CL molecular species was observed through a broad mass range (Fig. 1D, E and Fig. 2A). Over 50 distinct CL molecular species were readily identified which included polyunsaturated fatty acids such as arachidonic acid (C_20:4_) (AA), docosahexaenoic acid (C_22:6_) (DHA), and linoleic acid (C_18:2_) (LA) that are highly susceptible to peroxidation (Fig. 2B, C). The 18:1 fatty acid is predominantly composed of CL and represents 49.8% of CL compositions (Fig. 2B, C). Major other species of acyls are 20:4 (AA, 20.0%), 22:6 (DHA, 12.3%) and 18:2 (LA, 8.1%) (Fig. 2B, C). Quantitative assessments revealed a significant reduction of CL molecular ions with *m/z* (mass-to-charge ratio) 714.00, 727.00, 728.01, 736.99, 738.00, 739.00, 750.00, 751.00, 759.98, 760.99, 762.00, 763.00, 774.00, 796.99 as doubly-charged ions after SCI (Fig. 2A). About 21.4% of CL molecular species were significantly reduced at 3 h after SCI and 50% of CL molecular species was significantly reduced at 24 h after SCI. Acyls of CL such as 18:1, 20:4, 22:6 18:2, and 20:3 fatty acids were significantly reduced after SCI (Fig. 2B, C).

### Cardiolipin alteration induces neuronal death

**Fig. 3:**
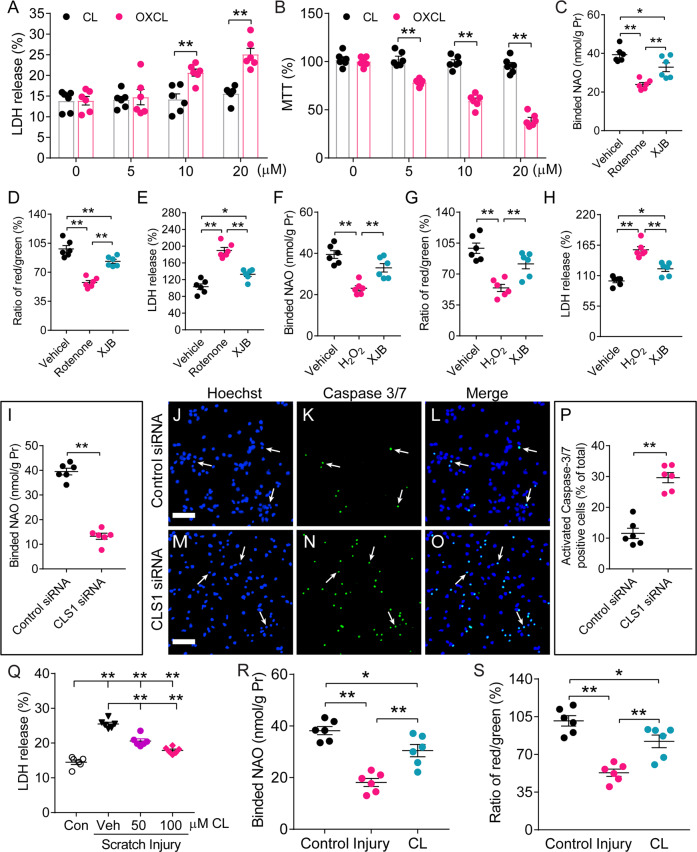
Effects of cardiolipin (CL) alteration on neuronal death. **A**, **B** Cultured spinal cord neurons were treated with the designated concentrations of oxidized CL (OXCL) and non-oxidized CL for 24 h. Neuronal cell death was measured by LDH, a stable cytoplasmic enzyme that is present in all cells but only released when the plasma membrane is damaged. MTT assay was used to determine the cell viability and mitochondrial activity, since tetrazolium is reduced to formazan by mitochondrial dehydrogenase activity. The oxidized CL induced spinal cord neuronal death (**A**) as measured by LDH release and mitochondrial dysfunction (**B**) as measured by MTT in a dose-dependent manner. **P < 0.01 (Two-way ANOVA, Tukey’s multiple comparisons test). Data represent the mean ± s.e.m. from 3 independent experiments. **C**–**H** The spinal neuronal cultures were exposed to rotenone (125 nM) or H_2_O_2_ (50 μM) either in the absence or presence of 10 μM XJB-5-131 (XJB) for 24 h. XJB was added 30 min before rotenone or H_2_O_2_ administration, and the culture medium was removed for LDH at 24 h after oxidative stress. XJB-5-131 reversed rotenone or H_2_O_2_-induced CL loss (**C**, **F**), mitochondrial dysfunction (**D**, **G**), and neuronal death (**E**, **H**). *P < 0.05, **P < 0.01 (One-way ANOVA, Tukey post hoc test, *n* = 6/group). Data represent the mean ± s.e.m. from 3 independent experiments. **I**–**P** Effects of CL synthase (CLS) siRNA on primary spinal neuronal death in vitro. Cultured spinal cord neurons were transfected with CLS1 siRNA (r) for 24 h. **I** CL content was assayed using acridine orange 10-nonyl bromide (NAO, Invitrogen). Knocking down CL synthase, the key enzyme of de novo CL biosynthesis, with CLS1 siRNA significantly decreased CL. **J**–**O** Activated caspase-3/7 was examined using CellEvent™ Caspase-3/7 Green Detection Reagent (Invitrogen). Apoptotic cells with activated caspase-3/7 showed bright green nuclei (arrows). Bar = 100 μm. **P** Bar graph showed that CLS1 siRNA significantly induced neuronal apoptosis, evidenced by increased number of activated caspase-3/7 cells. **P < 0.01 (Student *t*-test, *n* = 6/group). Data represent the mean ± s.e.m. from 3 independent experiments. **Q**–**S** Effects of exogenous CL on mitochondrial dysfunction and spinal neuronal death in the scratch injury model in vitro. Cultured spinal cord neurons were pre-incubated with CL liposomes for 30 min before scratch injury. **Q** Scratch injury-induced neuronal death was significantly reversed by exogenous CL in a dose-dependent manner. **R**, **S** Exogenous CL (100 µM) also significantly reversed scratch injury-induced CL loss (**R**) and decreased mitochondrial membrane potential (MMP) (**S**). Veh, vehicle; *P < 0.05, **P < 0.01 (One-way ANOVA, Tukey’s multiple comparisons test, *n* = 6/group). Data represent the mean ± s.e.m. from 3 independent experiments.

To explore whether SCI-induced CL alteration (peroxidation and loss) was sufficient to induce cell death, we first investigated the effect of CL peroxidation on cell death of spinal cord neurons. We found that exposure of primary neurons to oxidized cardiolipin resulted in dose-dependent cell death (Fig. 3A, B). Second, we examined the effects of H_2_O_2_, a ROS inducer, and rotenone, an endogenous mitochondrial ROS inducer on CL peroxidation, mitochondrial function, and cell death in cultured spinal cord neurons. CL content was detected with acridine orange 10-nonyl bromide (NAO, Invitrogen), which binds to CL with high affinity. The results showed that rotenone and H_2_O_2_ induced CL loss (Fig. 3C, F), mitochondrial dysfunction (Fig. 3D, G), and neuronal death (Fig. 3E, H). Importantly, such effects were reversed by XJB-5-131 (XJB), a novel mitochondria-targeted electron and reactive oxygen species (ROS) scavenger that reduces CL degradation and cell death [18, 23], suggesting that CL oxidation induced neuronal death. Last, we investigated the effect of reduced CL on spinal cord neuronal death by knocking down CL synthase, a key enzyme of de novo CL biosynthesis, with cardiolipin synthase 1 (CLS1) siRNA in vitro. The results showed that CLS1 siRNA reduced CL content (Fig. 3I) and increased apoptotic cells positively stained with activated caspase-3/7 in their nuclei (bright green, Fig. 3J–P). These findings suggest that CL alteration could induce neuronal death.

### Exogenous cardiolipin protects spinal neurons from mechanically-induced cell death in vitro

To further explore the role of CL alteration in SCI, we first investigated whether exogenous CL reduces mechanical injury-induced neuronal death in an in vitro SCI model of mechanical scratch injury in rat primary spinal cord neurons. This model is useful for investigating the effect of mechanical damage on neurons, such as what happened in a SCI, and for testing the neuroprotective effects of certain agents on mechanically injured neurons [24]. Our results showed that pre-treatment with CL liposomes reduced scratch-induced neuronal death in a dose-dependent manner (Fig. 3Q). At 24 h after the scratch injury, mitochondria were isolated from cultured spinal cord neurons for CL and mitochondrial function analysis. Our results showed that scratch injury induced CL loss (Fig. 3R) and reduced mitochondrial membrane potential (MMP) (Δψm) (Fig. 3S) in cultured spinal cord neurons. Importantly, such effects were significantly reversed by the administration of 100 µM exogenous CL (Fig. 3R, S).

### Oxidative stress induces mitochondrial cytosolic phospholipase A_2_ (cPLA_2_) activation, which mediates cardiolipin loss, leading to mitochondrial dysfunction and neuronal death after SCI

**Fig. 4:**
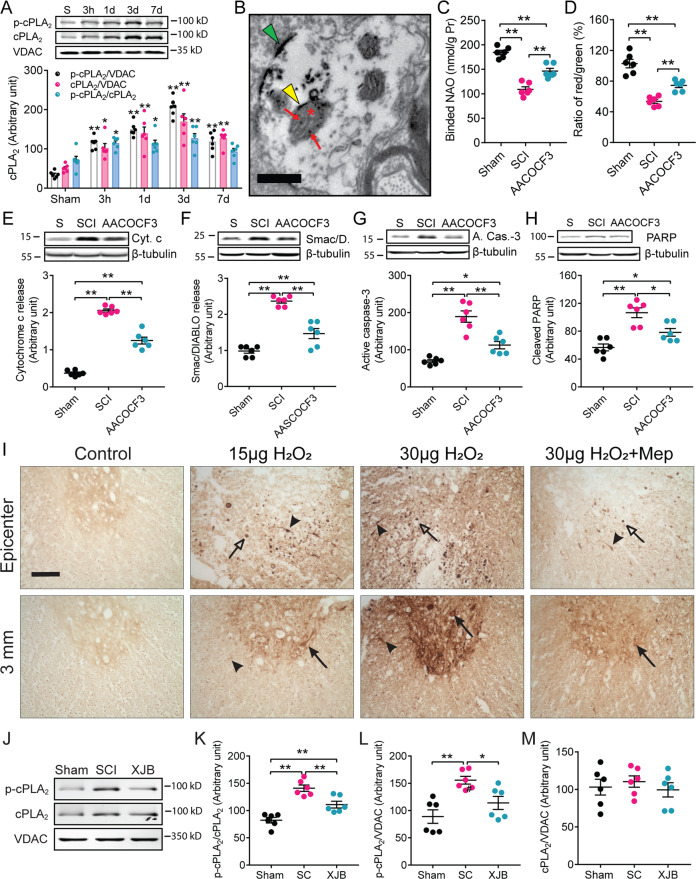
Oxidative stress induces cPLA_2_ activation and blockade of cytosolic phospholipase A_2_ (cPLA_2_) with AACOCF3 reduced cardiolipin (CL) loss, mitochondrial dysfunction, and apoptosis after spinal cord injury (SCI). **A** Time course of mitochondrial cPLA_2_ expression and activation after SCI. Data represent mean ± s.e.m., *P < 0.05, **P < 0.01 vs sham (Two-way ANOVA, Tukey’s multiple comparisons test, *n* = 6 rats/group). **B** An electron microscopic image shows phosphorylated cPLA_2_ (p-cPLA_2_) immunopositive peroxidase reaction products in a neuronal dendrite in the rat spinal ventral horn. p-cPLA_2_ immunoreactivity (IR) was observed to localize in mitochondria (*) including both the outer (yellow arrow) and inner (red arrows) mitochondrial membranes, and on the cell membrane (green arrow) at 3 days after SCI. Bar = 0.25 μm. **C** cPLA_2_ activation-induced CL loss was reversed by AACOCF_3_, a cPLA_2_ inhibitor. **D** SCI induced a decrease in the ratio of red/green in the MMP (Δψm) assay, which was reversed by AACOCF_3._ (**E**–**H**) AACOCF_3_, a cPLA_2_ inhibitor, reversed SCI-induced cytochrome c release (**E**), Smac/DIABLO release (**F**), active caspase-3 expression (**G**), and cleaved PAPRP expression (**H**). Data represent mean ± s.e.m. *P < 0.05, **P < 0.01 (One-way ANOVA, Tukey’s multiple comparisons test, *n* = 6 rats/group). Cyt. c cytochrome c, Smac/D. Smac/DIABLO, A. casp-3 active caspase-3. **I**–**M** Oxidative stress induces cPLA_2_ activation after SCI. **I** Immunohistochemistry shows a marked increase in p-cPLA_2_-expression, a marker of cPLA_2_ activation, at the injury epicenter and 3 mm rostral to it after a single injection of H_2_O_2_ into the T9 spinal cord. Right column images show that H_2_O_2_-induced p-cPLA_2_ expression was substantially reversed by mepacrine (5 mg/kg), a PLA_2_ inhibitor. The expression of p-cPLA_2_ was found in neurons (arrows), swelling axons (open arrows), and glial cells (arrowheads). Bar, 100 μm. **I**–**L** Administration of XJB (10 mg/kg, i.p.) at 30 min post-injury significantly reduced SCI-induced mitochondrial cPLA_2_ activation. **J** Representative images of phosphorylated cPLA_2_ (p-cPLA_2_), cPLA_2_, and VDAC expression. **K** Compiled results in a bar graph for the ratio of p-cPLA_2_/cPLA_2_ expression. **L** Compiled results in a bar graph for the ratio of p-cPLA_2_/VDAC expression. **M** Compiled results in a bar graph for the ratio of cPLA_2_/VDAC expression. *P < 0.05, **P < 0.01 (One-way ANOVA, Tukey’s multiple comparisons test, *n* = 6). Data represent the mean ± s.e.m.

**Fig. 5:**
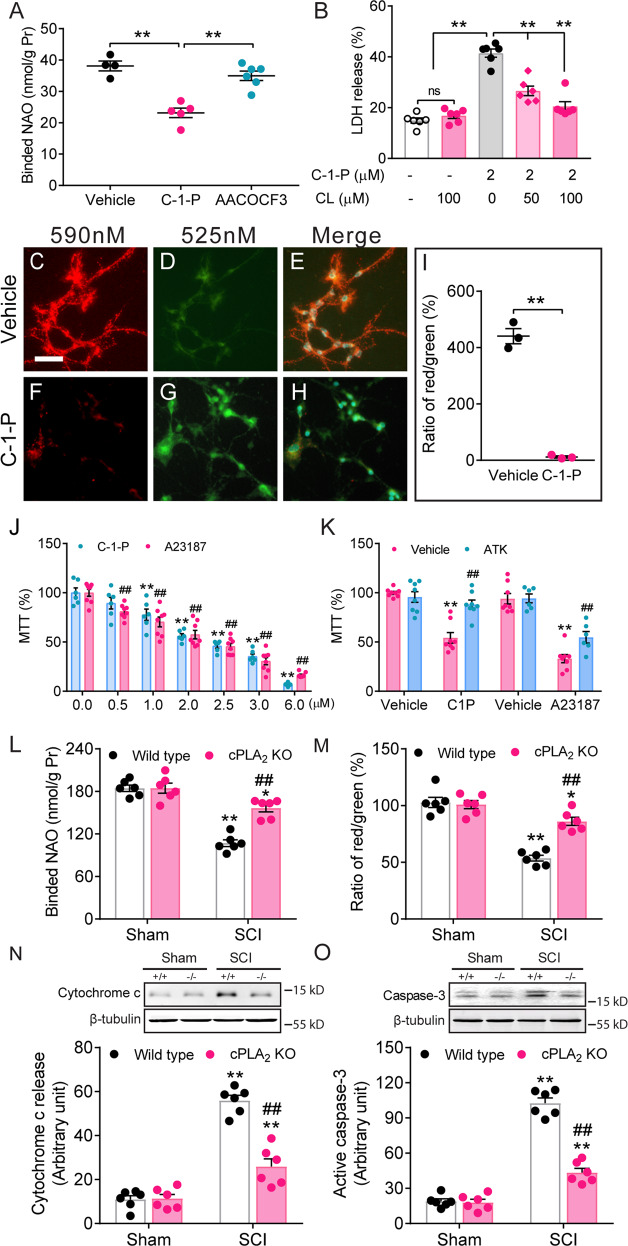
Cytosolic phospholipase A_2_ (cPLA_2_) activation induces cardiolipin (CL) loss, leading to mitochondrial dysfunction and neuronal death. **A** cPLA_2_ inhibitor AACOCF3 revised cPLA_2_ activation-induced CL loss. **P < 0.01 (One-way ANOVA, Tukey’s multiple comparisons test, *n* = 4–6). Data represent the mean ± s.e.m. from 3 independent culture experiments. **B** CL reduced cPLA_2_ activation-induced neuronal death. CL liposomes were added 30 min before C-1-P (2 µM) treatment. **P < 0.01; ns no significance. (One-way ANOVA, Tukey’s multiple comparisons test, *n* = 6). Data represent the mean ± s.e.m. from 3 independent culture experiments. **C**–**H** C-1-P induced mitochondrial membrane potential (MMP, Δψm) change measured with the cationic dye JC-1 in cultured spinal neurons. Vehicle-treated neurons showed strong J-aggregation (red). After C-1-P treatment, the majority of neurons showed green staining due to low Δψm. Bar, 50 µm. **I** Bar graph shows C-1-P induced a significant decrease in the ratio of red/green, indicating that activation of cPLA_2_ induced mitochondrial dysfunction (**P < 0.01, Student *t* test, *n* = 3). **J** Cultured spinal cord neurons were treated with the designated concentrations of C-1-P or A23187 for 24 h. MTT assay revealed that both cPLA_2_ activators, C-1-P and A23187, induced mitochondrial dysfunction and neuronal death in a dose-dependent manner. **P < 0.01, ##*P* < 0.01 versus the vehicle control (One-way ANOVA, Tukey’s multiple comparisons test, *n* = 6–8). Data represent the mean ± s.e.m. from 3 independent experiments. **K** Importantly, mitochondrial dysfunction and neuronal death induced by C-1-P (2 µM) or A23187 (5 µM) were significantly reversed by AACOCF3 (15 µM), a cPLA_2_ inhibitor. AACOCF3 was added 30 min before C-1-P or A23187 administration, and the culture cells were examined for MTT at 24 h after the activator treatment. **P < 0.01 versus the vehicle control, ##*P* < 0.01 versus the C-1-P or A23187 group (Two-way ANOVA, Tukey’s multiple comparisons test, *n* = 7–8. Data represent the mean ± s.e.m. from 3 independent experiments. **L**–**O** cPLA_2_ ablation protected against CL loss (**L**), mitochondrial dysfunction (**M**), and apoptosis (**N**–**O**) induced by SCI. **L** NAO-labeled cardiolipin expression at 1 day after SCI. **M** A ratio of red/green, indicating mitochondrial function. **N** Cytochrome c release at 1 day after SCI. **O** Expression of active caspase-3 at 1 day after SCI. Data represent mean ± s.e.m. *P < 0.05, **P < 0.01 vs sham; ##*P* < 0.01 vs WT (Two-way ANOVA, Tukey’s multiple comparisons test, *n* = 6 mice/group).

CL, a mitochondrial phospholipid, is a substrate of phospholipase A_2_ (PLA_2_) [13, 25, 26]. In physiological conditions, PLA_2_ hydrolysis is preferentially limited to oxidized acyl chains, including those of oxidized CL, and this is helpful for the overall membrane physiology within mitochondria [27]. However, when PLA_2_ is overactivated, such as in pathological conditions, the enzymatic activities are therefore elevated and even non-oxidized CL are subject to degradation by the enhanced hydrolysis. cPLA_2_ is the most important PLA_2_ isoform [9, 28]. To explore whether cPLA_2_ was involved in SCI-induced CL loss, we first isolated the mitochondrial fraction from the injured spinal cord in a rat contusive SCI model using a Focus Subcell fraction kit. Western blot analysis showed that cPLA_2_ expression and activation were increased in the mitochondrial fraction after SCI and peaked at 3 days (Fig. 4A). An immuno-electron microscopic image showed the localization of phosphorylated cPLA_2_ (p-cPLA_2_)-immunoreactivity in both outer and inner mitochondrial membranes (Fig. 4B). These findings suggest that mitochondrial cPLA_2_ may play a role in mediating CL loss. Our previous report showed that a ROS and radical inducer H_2_O_2_ induced cPLA_2_ activation in cultured spinal cord neurons [29]. We also found that microinjections of H_2_O_2_ at two doses (15 and 30 μg) into the normal rat spinal cord at T9 resulted in a marked increase in the expression of p-cPLA_2_, an activated form of cPLA_2_, in a dose-dependent manner (Fig. 4I). We further found that SCI-induced mitochondrial cPLA_2_ activation was effectively reversed by the novel ROS and radical scavenger, XJB-5-131 (Fig. 4J–M). These findings suggest that mitochondrial oxidative stress can trigger cPLA_2_ activation. To further confirm the role of cPLA_2_ in CL loss after SCI, we determined whether blocking cPLA_2_ reduced CL loss. Our in vitro experiments showed cPLA_2_ activation by a cPLA_2_ activator ceramide-1-phosphate (C-1-P) induced CL loss (Fig. 5A), which was substantially reversed by AACOCF_3_, a cPLA_2_ inhibitor (Fig. 5A). Remarkably, blocking cPLA_2_ pharmacologically with AACOCF_3_ at 30 min postinjury in rats or genetically deleting cPLA_2_ in mice significantly reduced CL loss after SCI (Fig. 4C, Fig. 5L). These findings suggest that mitochondrial cPLA_2_ may play a role in mediating CL loss. To further assess whether activation of cPLA_2_ mediates CL alteration-induced cell death after SCI, we determined whether blocking cPLA_2_ activation with the cPLA_2_ inhibitor AACOCF3 would reduce injury-induced CL loss, resulting in improved mitochondrial function and reduced neuronal death after a contusive SCI in adult rats. Our results showed that cPLA_2_ activation by both cPLA_2_ direct and indirect activators C-1-P and A23187, respectively, induced mitochondrial dysfunction and neuronal death (Fig. 5C–K). Such C-1-P- or A23187-induced mitochondrial dysfunction and neuronal death were significantly reversed by AACOCF3, a cPLA_2_ inhibitor (Fig. 5K). Importantly, pre-treatment with CL liposomes reduced C-1-P-induced neuronal death in a dose-dependent manner (Fig. 5B), suggesting that CL loss mediates cPLA_2_ activation-induced neuronal death. Most importantly, both pharmacological blockade in rats and genetic deletion of cPLA_2_ in mice significantly reduced mitochondria dysfunction (Figs. 4D, 5M), cytochrome c release (Figs. 4E, 5N), Smac/DIABLO release (Fig. 4F), active caspase-3 expression (Figs. 4G, 5O), and cleaved PARP expression (Fig. 4H) after SCI. These findings strongly suggest that cPLA_2_ mediates CL alteration-induced cell death and that such cell death is mediated through a mitochondrial apoptotic cascade.

### XJB-5-131 attenuates SCI-induced cardiolipin peroxidation, mitochondria dysfunction, and apoptotic neuronal death after SCI

**Fig. 6:**
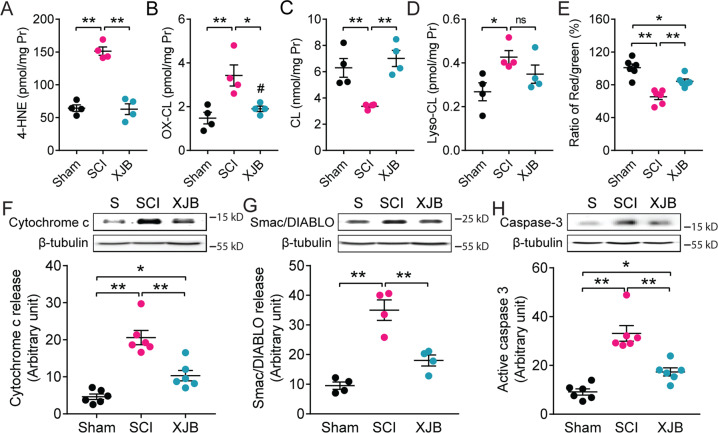
Cardiolipin (CL) peroxidation induced mitochondrial dysfunction and apoptosis. **A**–**D** lipidomic analysis at 1 day after SCI showed that XJB-5-131 (XJB, 10 mg/kg) attenuated SCI-induced mitochondrial 4-HNE expression (**A**), oxidized CL (**B**), CL loss (**C**), and lyso-CL (**D**) in adult rats. *P < 0.05, **P < 0.01 (One-way ANOVA, Tukey’s multiple comparisons test, *n* = 4 rats/group). Data represent mean ± s.e.m. **E**–**H** XJB attenuated mitochondrial dysfunction (**E**), cytochrome c release (**F**), Smac/DIABLO release (**G**), and active caspase-3 expression (**H**) at 1 day after SCI in adult rats. Data represent mean ± s.e.m. *P < 0.05, **P < 0.01 (One-way ANOVA, Tukey’s multiple comparisons test, *n* = 4–6 rats/group).

Mitochondrial oxidative stress not only induced CL peroxidation, but also stimulated cPLA_2_ activation. XJB-5-131 is a novel mitochondria-targeted ROS, electron, and radical scavenger which can cross the BBB and prevent the peroxidation of CL [18, 30–33]. To further explore the role of CL peroxidation in SCI, we tested whether attenuating CL peroxidation with XJB-5-131 would reduce mitochondrial dysfunction and apoptotic neuronal death after SCI. Lipidomic analysis showed that administration of XJB-5-131 (10 mg/kg, i.p.) at 30 min post-injury significantly reduced SCI-induced mitochondrial peroxidation (Fig. 6A), CL oxidation (Fig. 6B), CL loss (Fig. 6C), and lyso-CL (Fig. 6D) at 1 day after SCI. Our studies further showed that XJB-5-131 treatment significantly reduced SCI-induced mitochondrial dysfunction (Fig. 6E), cytochrome c release (Fig. 6F), Smac/DIABLO release (Fig. 6G), and active caspase-3 expression (Fig. 6H) at 1 day after SCI.

### XJB-5-131 reduces tissue damage and improved behavioral recovery after SCI

**Fig. 7:**
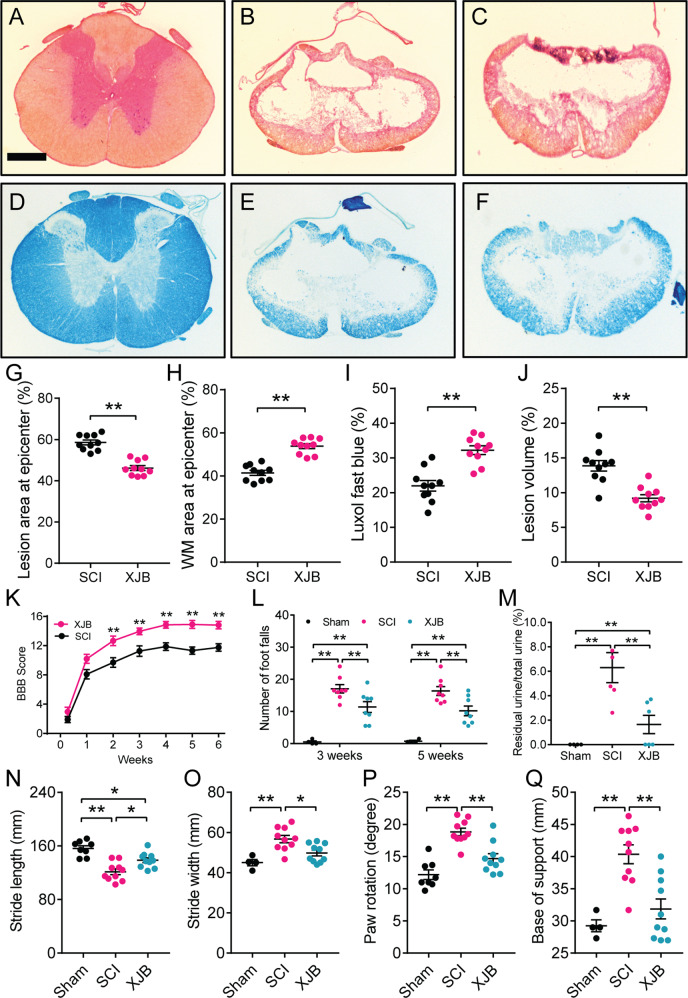
XJB-5-131 (XJB), a novel mitochondria-targeted ROS, electron, and radical scavenger, reduced tissue damage and improved behavioral recovery after a contusive SCI. **A**–**F** Representative sections show the lesion epicenter stained with cresyl violet and eosin (**A**–**C**) or with Luxol fast blue (**D**–**F**). XJB treatment (10 mg/kg) significantly reduced lesion area by 21.2% (**A**–**C**, **G**, **P < 0.01), increased white matter (WM) sparing by 30.0% (**A**–**C**, **H**, **P < 0.01), enhanced myelin sparing by 46.4% (**D**–**F**, **I**, **P < 0.01), and reduced lesion volume by 33.8% (**A**–**C**, **J**, **P < 0.01). Bar = 500 μm. **G**–**J** Student *t* test, (*n* = 10 rats/group). **K** Administration of XJB significantly improved Basso, Beattie, and Bresnahan (BBB) locomotor scores up to 6 weeks post-SCI in adult rats. **P < 0.01 vs vehicle-treated groups (Repeated measures ANOVA, Bonferroni’s multiple comparisons test, *n* = 10 rats/group). **L** XJB significantly reduced foot falls at 3 and 5 weeks post-injury. **P < 0.01 (Repeated measures ANOVA, Tukey’s multiple comparisons test, *n* = 4–10 rats/group). **M** XJB treatment significantly reduced residual urine in the urinary bladder at 2 weeks post-SCI. **P < 0.01 (One-way ANOVA, Tukey’s multiple comparisons test, *n* = 4–6 rats/group). **N**–**Q** XJB-5-131 also significantly increased stride lengths (**N**), decreased stride width (**O**), paw rotation angles (**P**), and base of support (**Q**) in the foot print analysis at 6 weeks post-injury as compared to the vehicle-treated SCI. *P < 0.05, **P < 0.01 (one-way ANOVA, Tukey’s multiple comparisons test, *n* = 4–10 rats/group). In **G**–**Q**, error bars represent mean ± standard error of the mean.

To determine whether inhibition of CL peroxidation promotes functional recovery, an array of behavior tests was performed on consecutive days following SCI to evaluate motor and sensorimotor functions. There were statistically significant effects of treatment group (F_(1, 18)_ = 15.77, *P* = 0.0009), test day (F_(6, 108)_ = 259.2, *P* < 0.0001), and the interaction of treatment group and test day (F_(6, 108)_ = 2.78, *P* = 0.0149; repeated measures ANOVA) for the Basso, Beattie, and Bresnahan (BBB) open field locomotion. At the first time point, 3 days after SCI, no significant difference was found between the two groups (Supplementary Fig. 2, Fig. 7K). From 2 weeks post-SCI, XJB-5-131 treatments significantly improved BBB scores for up to 6 weeks (Fig. 7K; *P* < 0.01). Repeated measures ANOVA also revealed that there were statistically significant effects of XJB-5-131 treatment (Fig. 7L; F_(2, 21)_ = 58.14, *P* < 0.0001) for grid walking at 3 and 5 weeks post-SCI. XJB-5-131 treatment significantly reduced residual urine in the urinary bladder at 2 week post-SCI (Fig. 7M; *P* < 0.01), suggesting that inhibition of CL peroxidation improved bladder function. Footprint analysis showed that administration of XJB-5-131 significantly improved the stride length (Fig. 7N; *P* < 0.05), stride width (Fig. 7O; *P* < 0.05), paw rotation angle (Fig. 7P; *P* < 0.01), and base of support (Fig. 7Q; *P* < 0.01) at 6 weeks post-SCI.

Because we demonstrated that administration of XJB-5-131 significantly improved behavioral recovery after SCI, we next examined whether such a treatment also would result in tissue protection in vivo. To ensure that the entire rostrocaudal expansion of the lesion was examined, a 1.5 cm-long cord segment was serially sectioned. Measurements of percentage total lesion volume, lesion area, and white matter sparing area were made from cresyl violet–stained and eosin-stained transverse sections spanning the entire lesion. Comparison of the lesion area at the injury epicenter demonstrated that XJB-5-131 treatments resulted in a significant reduction of lesion area by 21.2% (Fig. 7A–C, G; *P* < 0.01). The reduction in lesion area was accompanied by a corresponding increase in the area of white matter sparing by 30.0% (Fig. 7H; *P* < 0.01) at 6 weeks post-SCI. In addition, Luxol fast blue staining showed that the XJB-5-131 treatment resulted in a corresponding increase in myelin sparing by 46.4% (Fig. 7D–F, I; *P* < 0.01). Finally, stereological assessments of the lesion volume showed that XJB-5-131 treatment resulted in a significant reduction in the percentage of total lesion volume by 33.8% (Fig. 7J; *P* < 0.01).

## Discussion

**Fig. 8:**
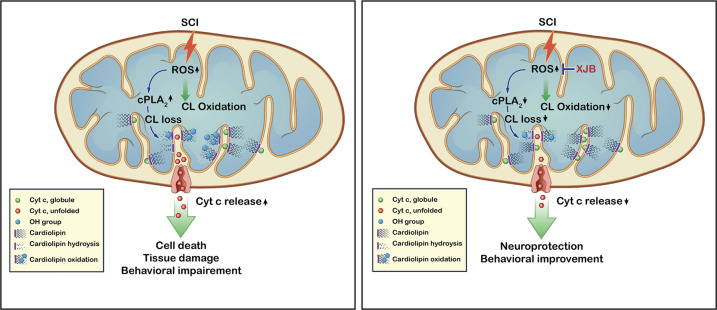
Role of cardiolipin (CL) in spinal cord injury (SCI). Schematic drawing shows that SCI-induced CL alterations (peroxidation and loss) and downstream cascades leading to apoptotic cell death and functional impairment. Under normal circumstances, cardiolipin binds and retains cytochrome c (cyt c) in its native molten-globule structure at the inner mitochondrial membrane, keeping it close to the electron transport chain. (Left) During SCI, however, a massive production of ROS leads to accumulation of unfolding of cyt c, enhancing cyt c’s peroxidase activity, which targets polyunsaturated acyl chains of CL and induces CL peroxidation. ROS also induces cPLA_2_ activation, which hydrolyzes CL and induces CL loss. Oxidation and loss of CL weakens and decreases its interaction with cyt c, which is now released and escapes to the cytosol via permeabilization of the outer mitochondrial membrane, triggering apoptosis and functional impairment. (Right) Treating SCI with XJB-5-131 (XJB), a novel mitochondria-targeted antioxidant, blocks CL alteration, attenuates apoptotic cell death, and improves functional recovery.

To our knowledge, this is the first study demonstrating alteration of CL after SCI using mass spectrometry-based lipidomics and the role of CL alteration in SCI as illustrated in Fig. 8. We identified over 50 distinct CL molecular species. Among them, 50% were significantly reduced after SCI. The reduced CL species contained mainly polyunsaturated fatty acids such as AA, DHA, and LA that are highly susceptible to peroxidation. Our results showed that in addition to inducing CL oxidation, mitochondrial oxidative stress also activated cPLA_2_ which led to a further loss of CL by acyl chain hydrolysis. We demonstrated that both CL peroxidation and CLS siRNA-induced CL loss resulted in neuronal apoptosis. Administration of CL reduced injury-induced spinal cord mitochondrial dysfunction and neuronal death. We showed that mitochondrial cPLA_2_ expression and activation were markedly increased after SCI, which was located in the inner and outer mitochondrial membranes. Activated cPLA_2_ induced CL loss, leading to mitochondrial dysfunction and neuronal apoptosis after SCI. Notably, treatment with the mitochondrial-target ROS scavenger XJB-5-131 reduced CL peroxidation and loss, leading to decreases of mitochondrial dysfunction and neuronal apoptosis after SCI. Most importantly, we provide anatomical and functional evidence showing that XJB-5-131 significantly reduced cell death and tissue damage, and improved behavioral recovery after SCI. These findings collectively suggest that CL alteration could be an attractive therapeutic target for increasing neuronal survival and promoting recovery of function after SCI.

Female animals are routinely used in SCI studies because female animals allow for easier manual expression of bladders after SCI, less urinary tract infection, and less mortality [34–37]. In addition, we and others have shown that no significant differences were detected in histological and behavioral outcomes between male and female animals after SCI [38–40]. Therefore, female animals were used in this study.

### Cardiolipin alteration occurs at an early stage after SCI triggering an apoptotic cascade

A decrease in the content of mitochondrial CL is the most frequently reported pathological alteration of the CL profile [13]. In our study, increases of biomarkers of apoptosis (cytochrome c release, Smac/DIABLO release, and active caspase-3 expression) at the early stage of SCI suggested an early CL alteration post-SCI. CL alteration has been reported to cause the release of cytochrome c and Smac/DIABLO and to activate caspase-3 during induction of apoptosis [16, 20, 21]. In our SCI model, the release of cytochrome c and Smac/DIABLO preceded active caspase-3 expression, which was consistent with a previous report that CL oxidation preceded caspase-3 activation after traumatic brain injury [17]. The decrease of CL at 3 and 24 h after SCI, shown by the lipidomic analysis, further confirmed that CL alteration is an early event after SCI. A corresponding increase in lyso-CL at only 24 h post-injury indicated that SCI induced CL degradation at 24 h after SCI. Decreases in CL content are often used as an indicator of CL oxidation because CL oxidation may lead to an overall loss of detectable CL content, either by preferential hydrolysis of peroxidized acyl chains by PLA_2_, direct decomposition of lipid peroxides, or the formation of CL-protein complexes that would no longer be detected as phospholipids [13]. Lipid peroxidation increased at 3 and 24 h after SCI (Fig. 1F), suggesting that CL peroxidation may occur at 3 and 24 h after SCI. After SCI, 50% of CL species were significantly reduced and the reduced CL species contained mainly polyunsaturated fatty acids that are highly susceptible to peroxidation. Additionally, 4-HNE, a lipid peroxidation marker, also increased after SCI. CL is a likely early target of ROS attack in mitochondria due not only to its high content of unsaturated fatty acids, but also to its location in the IMM near the sites of major ROS production [41]. A change in the oxidative state or content of CL has been proposed to trigger the mitochondrial switch from ATP generation to apoptosis initiation [13, 14, 42]. Thus, these results suggest that SCI induces early CL peroxidation, degradation, and loss, triggering an apoptotic cascade.

### Cardiolipin loss is mediated by phospholipase A_2_

Although CL content was significantly decreased after SCI, the mechanism(s) by which it was decreased remains unclear. Under normal conditions, PLA_2_ preferentially hydrolyzes oxidized phospholipids including oxidized CL to maintain mitochondrial membrane stability and function [27]. Under pathological conditions, over-activated PLA_2_ can directly induce CL loss through enhanced hydrolysis and degradation of CL, resulting in mitochondrial dysfunction. This can trigger an apoptotic signaling cascade, eventually leading to cell death and tissue damage. The phospholipases A_2_ can be classified into three major enzyme families: cytosolic PLA_2_ (cPLA_2_), secretory PLA_2_ (sPLA_2_), and Ca^2+^-independent PLA_2_ (iPLA_2_) [3, 9, 43]. Mammalian phospholipases, including cPLA_2_, sPLA_2_, and iPLA_2_, are reported to have CL-hydrolyzing activities [25, 26], and several studies suggest that iPLA_2_ could be a candidate catalyst capable of hydrolyzing CL in mitochondria [44–49]. However, which PLA_2_ subtype is involved in CL hydrolysis after SCI, or its preference for peroxidized CL, remains to be elucidated. Cytosolic PLA_2_ is the most important PLA_2_ isoform because it has been implicated as an effector in the receptor-mediated release of AA and exhibits a strong preference for deacylation of AA over other fatty acids [4, 50]. Our in vitro experiments showed that activated cPLA_2_ induced CL loss, leading to mitochondrial dysfunction and neuronal death. Such effects, however, were substantially reversed by AACOCF3, a cPLA_2_ inhibitor. Our in vivo experiments further showed that the expression and activity of cPLA_2_ significantly increased in the mitochondria following SCI. Immuno-EM analysis revealed that activated cPLA_2_ was expressed in the mitochondria. Remarkably, blocking cPLA_2_ pharmacologically with AACOCF3 reduced CL loss, and alleviated mitochondrial dysfunction and neural death after SCI. Furthermore, genetic deletion of cPLA_2_ reduced CL loss, resulting in neuroprotection after SCI. Taken together, these findings suggest that SCI-induced CL loss, at least part, is mediated by cPLA_2_ activation.

### Cardiolipin alteration results in mitochondrial dysfunction and neuronal death

CL is a mitochondrial-specific phospholipid localized in the IMM where it is required for optimal mitochondrial function [13]. It has been reported that CL is the only phospholipid in mitochondria that undergoes early oxidation after injury [17] or during apoptosis [16]. The oxidation is catalyzed by a CL-specific peroxidase activity of CL-bound cytochrome c [16]. Furthermore, oxidized CL is required for the release of proapoptotic factors [16]. Our results showed that decreasing CL by knocking down the CL synthase (CLS1) induced neuronal apoptosis, and pre-treatment with CL liposomes reduced scratch-induced mitochondrial dysfunction and neuronal death, suggesting that CL alteration may mediate mitochondrial dysfunction and neuronal death after SCI. Our in vivo experiments further showed that pharmacological blocking or genetic deletion of cPLA_2_ in mice attenuated CL loss, leading to improvement of mitochondrial function and neuronal survival. One mechanism of CL alteration-induced neuronal death could be its interaction with cytochrome c. CL electrostatically anchors cytochrome c to the IMM and plays an important regulatory role in cytochrome c release, which triggers the downstream events in apoptosis [13, 16, 30]. CL peroxidation and/or loss weakens its interaction with cytochrome c, which is then released and escapes to the cytosol via the mitochondria transition pore formed by the proapoptotic Bcl-2 family proteins, triggering apoptosis [13, 16, 30].

### Pharmacological inhibition of CL alteration results in neuroprotection and functional recovery after SCI

A significant finding of this study is that pharmacological inhibition of CL alteration with XJB-5-131 reduced mitochondrial dysfunction, neuronal death, and tissue damage, and promoted functional recovery in adult rats after SCI. Our results showed that XJB-5-131 not only inhibited CL peroxidation after SCI, but also reduced CL loss by inhibition of cPLA_2_ activation after SCI. Several studies show that CL content can be preserved when ROS is reduced in ischemic heart and skeletal muscle [51–54]. Apoptosis has been considered as a key mechanism of cell death following SCI [55–57]. Notably, administration of XJB-5-131 at 30 min post-injury significantly reduced SCI-induced biomarkers of apoptosis including cytochrome c release, Smac/DIABLO release, and active caspase-3 expression. Importantly, our post-treatment results showed a long beneficial effect on anatomical and functional improvements. In agreement with our observation, XJB-5-131 inhibited traumatic brain injury (TBI)-induced CL alteration, had potent neuroprotective activity in vitro, reduced cortical lesion volume, and ameliorated behavioral deficits after TBI [18]. XJB-5-131 also reduced oxidative DNA damage, improved mitochondrial function, enhanced neuronal survival, and suppressed motor decline in a mouse model of Huntington’s Disease [58]. Our results suggest that CL alteration contributes to the pathogenesis of SCI and that targeting the CL alteration could be a promising therapeutic strategy for intervention after SCI.

In conclusion, we demonstrated that SCI induced significant CL alteration (peroxidation and loss) at an early stage of SCI, and that such alterations induced mitochondrial dysfunction and neuronal death, ultimately, leading to tissue damage and functional deficits. Notably, the mitochondrial-target ROS scavenger XJB-5-131 treatment reduced CL peroxidation and loss, leading to decreases of mitochondrial dysfunction and neuronal apoptosis after SCI. Remarkably, pharmacologic inhibition of CL alteration with XJB-5-131 reduced tissue damage and ameliorated behavioral deficits after SCI in rats. These findings suggest that CL alteration could be a novel mechanism that mediates injury-induced neuronal death, and therefore represents a potential therapeutic target for ameliorating secondary SCI.

## Materials and methods

### Reagents

Methyl-tert-butyl ether (Fisher Scientific, Fair Lawn, NJ), Methanol (Burdick and Jackson, Muskegon, MI), Millipore deionized water (Milli-Q Advantage A10, EMD Millipore, Billerica, MA), Isopropanol (Burdick and Jackson, Muskegon, MI), Lithium hydroxide (Sigma-Aldrich, St. Louis, MO). 1,1’,2,2’-Tetramyristoyl cardiolipin (T14:0 CL) (Avanti Polar Lipids, Inc., Alabaster, AL), (±)−4-hydroxy-9,9,9-d3-non-2E-enal (d3-4-HNE) (Cayman Chemical, Ann Arbor, MI). All other chemicals used in this study were from Sigma–Aldrich (St. Louis, MO) except for those specifically indicated.

### Animals

Female Sprague-Dawley rats (210–230 g) were purchased from Harlan (Indianapolis, IN). Female cPLA_2_^−/−^ mice and wild-type (WT) littermates (12 weeks, 18–24 g) generated from heterozygous breeding pairs were used in this study. Breeding pairs of male and female heterozygous (cPLA_2_^+/−^) mice were kindly provided by Dr. J. Bonventre (Harvard Medical School). The breeding was carried out at Indiana University School of Medicine Laboratory Animal Resource Center. All cPLA_2_ mice were on a C57/BL6 background [28]. The animals were maintained on a 12 h/12 h light/dark cycle with food and water freely available. All surgical interventions, treatments, and postoperative animal care were performed in accordance with the Guide for the Care and Use of Laboratory Animals (National Research Council) and the Guidelines of the Institutional Animal Care and Use Committee of the Indiana University School of Medicine.

### Contusive spinal cord injury and treatment

Animals were randomly divided into the designated groups. A contusive SCI in rats was performed at the 10th thoracic (T10) vertebral level using an Infinite Horizon Impactor (Infinite Horizons, Lexington, KY) at an impact force of 175 kdyne according to a previously published method [59]. The acceptable injury displacement range was 775–899 µm and any rats with an injury displacement smaller or larger than that range were excluded from the study. The mice underwent a T10 contusive SCI using the same Infinite Horizon Impactor (Infinite Horizons) at an impact force of 60 kdyne as was described previously [28, 60]. The acceptable injury displacement range was 375–475 µm and any mice with an injury displacement smaller or larger than that range were excluded from the study. For the sham-operated controls, the animals underwent a T10 laminectomy without the impact. After injury, muscle and skin at the site of exposure were closed in layers, and animals were placed in the cage on a heating pad until full recovery from anesthesia. Manual bladder expression was carried out at least twice times daily until reflex bladder emptying was established. For the 1 day treatment experiment, rats were injected with XJB (10 mg/kg, i.p.) or vehicle at 30 min after SCI. XJB-5-131 was kindly provided by Dr. Peter Wipf (Department of Chemistry, University of Pittsburgh, Pittsburgh, PA). At 1 d post-injury, rats were sacrificed and spinal cord segments (10 mm) containing the injury epicenter were removed for lipidomics, mitochondrial dysfunction, and Western blot analysis. In subgroups, rats were treated i.p. with AACOCF_3_ (15 mg/kg, Cayman Chemicals, Ann Arbor, MI) or vehicle at 30 min post-injury. Rats were sacrificed at 1 d post-injury, and spinal cord segments containing the injury epicenter were removed for biochemical and Western blot analysis. We chose the 1 day time point for these measurements because these early events such as lipid peroxidation [61, 62], cPLA_2_ activation (Fig. 4A), and apoptosis [63] either peaked or near peaked at this time point after SCI. For behavioral and histological assessments, rats were treated with XJB (10 mg/kg, i.p.) or vehicle, administered at 30 min after SCI, and then daily for 6 days. Behavioral tests were performed at the designated times post-injury. At the 6th week post-injury, rats were sacrificed and their tissues processed for histology.

### Lipid extraction and preparation

Lipids from spinal cord segments (10 mm) were extracted as described previously [64, 65]. Rat spinal cord samples (100 mg) were homogenized in 0.5 ml 10 x diluted PBS in 2.0-ml cryogenic vials (Corning Life Sciences, Tewksbury, MA) by using a digital sonifier (Branson 450, Danbury, CT). Protein assay on the homogenates was performed by using a bicinchoninic acid protein assay kit (Thermo Scientific, Rockford, IL) with bovine serum albumin as standards. All determined lipid levels were normalized to the protein content of individual samples.

Individual homogenate of the spinal cord samples (equal ~0.5 mg protein amount) was accurately transferred into a disposable glass culture test tube. An internal standard mixture including T14:0 CL and d3-4-HNE for quantitation of all reported lipid classes was added prior to lipid extraction based on protein concentration. These internal standards were selected because they represent 1% of endogenous cellular lipid molecular species present as demonstrated by ESI/MS lipid analysis without the addition of these internal standards. Lipids were extracted by methyl-tert-butyl ether [65]. Each lipid extract was resuspended in a volume of 400 μl of CHCl_3_/MeOH (1:1, v/v) per mg of protein, flushed with N_2_, capped, and stored at −20 °C for electrospray ionization mass spectrometry (ESI-MS) analysis.

### Electrospray ionization mass spectrometry-based lipidomic analysis

Electrospray ionization mass spectrometry analysis was performed according to our protocol as described previously [64]. For ESI direct infusion analysis, lipid extract was further diluted to a final concentration of ~500 fmol/µL by CHCl_3_/MeOH/isopropanol (1/2/4, v/v/v) with or without 0.02% (v/v) LiOH-saturated MeOH solution, and the mass spectrometric analysis was performed on a QqQ mass spectrometer (Thermo TSQ VANTAGE, San Jose, CA) or a Q-Exactive mass spectrometer (Thermo TSQ VANTAGE, San Jose, CA) equipped with an automated nanospray device (TriVersa NanoMate, Advion Bioscience Ltd., Ithaca, NY) and operated with Xcalibur software. Identification and quantification of lipid molecular species were performed using an automated software program [66].

### Mitochondrial and cytosolic fractions isolation

Mitochondrial- and cytosolic-enriched fractions were isolated from fresh spinal cord tissue using the Focus SubCell kit (G-Biosciences, St. Louis, MO) according to the manufacturer’s protocol. In brief, 100 mg of fresh spinal cord tissue was homogenized and centrifuged at 700 × *g* for 10 min to pellet nuclei. The supernatant was removed and centrifuged at 12,000 × *g* for 15 min to pellet mitochondria. The supernatant was removed from the mitochondrial pellet for a cytosolic fraction. The protein content of all samples was assessed by the Bradford method (Bio-Rad Protein Assay, Hercules, CA) and normalized prior to adding sample buffer.

### Western blotting

Western blot analysis was performed according to our protocol as described previously [28, 59]. Briefly, for equal protein concentration from each sample was loaded onto polyacrylamide gel, separated by SDS-PAGE, and transferred to a nitrocellulose membrane by electrophoresis. The membrane was blocked in Odyssey blocking buffer for 1 h at room temperature. The primary antibodies were added to the membrane and incubated overnight at 4 °C. The primary antibodies included rabbit polyclonal anti-caspase-3 antibody (Cat# 9664, cleaved, 1:1000, 0.04 µg/ml, Cell Signaling Technology, Boston, MA), mouse anti-cytochrome c antibody (Cat# 556433, 1:300, 1.67 µg/ml, 7H8.2C12, BD Pharmingen, San Jose, CA), mouse anti-Smac/DIABLO antibody (Cat# 612246, 1:1000, 0.25 µg/ml, BD Transduction Laboratories, San Jose, CA), rabbit anti-PARP-1 (Cat# 9542, 1:500, 0.0976 µg/ml, Cell Signaling Technology, Boston, MA), rabbit anti-Bcl-2 (Cat# sc-492, 1:100, 2 µg/ml, Santa Cruz Biotechnology, Santa Cruz, CA), rabbit anti-Bax (Cat# sc-526, 1:100, 2 µg/ml, Santa Cruz Biotechnology, Santa Cruz, CA), and mouse anti-β-tubulin antibody (Cat# 5293, 1:1000, 4.7 µg/ml, Sigma, St. Louis, MO). For mitochondrial cPLA_2_ expression, primary antibodies included mouse monoclonal anti-cPLA_2_ antibody (Cat# sc-454, 1:100, 2 µg/ml, Santa Cruz Biotech, CA), polyclonal rabbit anti-phospho-cPLA_2_ antibody (Cat# 2831, 1:500, 0.356 µg/ml, Cell Signaling, Boston, MA), and rabbit anti-VDAC antibody (Cat# 4661, 1:1000, 2.6 µg/ml, Cell Signaling, Boston, MA). The membrane was washed 4 times for 5 min with PBS-T (PBS + 0.1% Tween-20) at room temperature, incubated with a secondary IRDye 800CW goat anti-rabbit (Cat# 926-32211, 1:5000, 0.2 µg/ml, LI-COR Biosciences, Lincoln, NE) or IRDye 680RD goat anti-mouse (Cat# 926-68070, 1:5000, 0.2 µg/ml, LI-COR Biosciences, Lincoln, NE) for 1 h, then washed 3 times for 5 min with PBS-T and twice for 5 min with PBS. The Western blot was imaged and quantified using a Li-Cor Odyssey Infrared Imaging system (LI-COR Biosciences) according to the manufacturer’s instruction. For the negative control, primary antibodies were omitted.

### Preparation of cardiolipin liposomes

Cardiolipin (Avanti Polar Lipids, Inc.) from bovine heart, stored in chloroform, was dried under nitrogen. Then CL was mixed in vortex in phosphate-buffered saline (PBS). After completion of hydration, the milky suspension was transferred to an Avanti^®^ Mini-Extruder (Avanti Polar Lipids, Inc.) and extruded through a series of polycarbonate membranes down to a pore size of 0.1 µm. Liposomes were used immediately after preparation.

Oxidized CL preparation followed procedures described previously [67] with modification. The milky suspension made above was further sonicated with a bath-type sonicator (Branson 1510, Ultrasonics Corporation, Danbury, CT) for 5 min to make opalescent stable vesicles. The resultant vesicular preparation was flushed for 2 min with 100% oxygen gas, tightly sealed, and kept at 37 °C for 24 h. CL oxidation was measured with Peroxide Assay Kits (cat:23285, Thermo Scientific Pierce, Rockford, IL) according to the manufacturer’s instructions. The oxidized CL vesicles were transferred to an Avanti^®^ Mini-Extruder (Avanti Polar Lipids, Inc.) and extruded through a series of polycarbonate membranes down to a pore size of 0.1 µm. Liposomes were used immediately after preparation.

### Spinal cord neuronal culture, cell treatment and viability assessment

Cells were obtained from embryonic (E) day 14 rat spinal cords by gentle trituration according to our previously described protocol [28, 59, 68, 69]. Under this culture condition, a purity of >85% spinal cord neuronal population was obtained on the seventh day in vitro (Supplementary Fig. 3). In this experiment, cultured spinal cord neurons were exposed to oxidized (OXCL) and non-oxidized cardiolipin (CL) liposomes at 3 hypothetical concentrations (5, 10, and 20 µM). The cultures were maintained for an additional 24 h for MTT assay using a CellTiter 96® Non-Radioactive Cell Proliferation Assay kit (Promega Corporation, Madison, WI) or the culture medium was removed for lactate dehydrogenase (LDH) release assay using a CytoTox 96 Non-Radioactive Cytotoxicity Assay kit (Promega). For cardiolipin oxidation experiments, **t**he spinal neuronal cultures were exposed to H_2_O_2_ (50 μM, Sigma) or Rotenone (0.125 μM, Sigma) either in the absence or presence of 10 μM XJB for 24 h. Cytosol and mitochondria of these cells were isolated for assessment of cardiolipin content, mitochondrial dysfunction, cytochrome *c* release, and Smac/DIALO release. Cell death was examined by LDH releasing assay. For cPLA_2_ experiments, cultures were then treated with the designated concentration of Ceramide-1-phosphate (C-1-P), A23187, and/or AACOCF3 or cardiolipin for designated times. The cultures were maintained for an additional 24 h for MTT assay or the culture medium was removed for LDH release assay. In a subset of cultures, spinal cord neurons were used for the mechanical scratch injury model, mitochondrial potential, and cardiolipin content analysis.

### Mechanical scratch injury in cultured spinal cord neurons and CL treatment

An in vitro model of mechanical injury in rat primary spinal cord neurons is useful for investigating the precise effect of mechanical damage on neurons excluding other contributing factors, and to detect the neuroprotective effect of certain factors on mechanically injured neurons [24]. Scratch injury in cultured spinal cord neurons was performed according to a previous publication [24]. Briefly, cell bodies and processes were cut mechanically with a cataract knife in 24-well culture plates for cell death and 10 cm dishes for mitochondrial studies (Supplementary Fig. 4). In order to standardize the damage in every well, the self-made cardboard painting grid with 2 mm intervals was placed underneath the transparent plastic plates. After this, the blade was allowed to move along the gridlines slowly and lightly during scratching. At 24 h after scratch injury, mitochondria were isolated from cultured spinal cord neurons for CL and mitochondrial function analysis. CL was assayed with acridine orange 10-nonyl bromide (NAO, Invitrogen), a highly specific probe of CL, which has been used to determine CL levels as described previously [70–72]. Mitochondrial membrane potential (MMP, Δψm) was determined using a cationic dye JC-1 (Invitrogen) loaded in isolated mitochondria. In a subset of cultures, scratched cultures were treated with the designated concentration of CL liposome for 24 h and the culture medium of each well was removed for lactate dehydrogenase (LDH) release assay using a CytoTox 96 Non-Radioactive Cytotoxicity Assay kit (Promega).

### Enrichment of neuron mitochondria with cardiolipin

Cultured spinal cord neurons were pre-incubated with CL liposomes (50 and 100 µM) for 30 min before scratch injury or C-1-P (2 µM) treatment. The cultures were maintained for an additional 24 h and the culture medium of each well was removed for LDH release assay using a CytoTox 96 Non-Radioactive Cytotoxicity Assay kit (Promega Corporation, Madison, WI). In a subset of cultures, 24 h after scratch injury with pre-treatment of 100 µM CL, mitochondria were isolated from cultured spinal cord neurons for CL and mitochondrial function analysis.

### Mitochondrial membrane potential (MMP, Δψm) assay

Mitochondrial membrane potential was assessed using the JC-1 Mitochondrial Membrane Potential Assay kit (Cayman Chemical Company Ann Arbor, Michigan) according to the manufacturer’s instructions. Briefly, mitochondria (10 µg), isolated from the fresh spinal cord tissue or spinal cord neuron culture, were incubated in 100 μl standard incubation medium (125 mM KCl, 3 mM KH2PO4, 0.5 mM MgCl2, 3 mM glutamate, 3 mM succinate, 0.1% BSA, 10 mM HEPES, pH 7.4) with 10 μl of the JC-1 staining solution at 37 °C for 15 min. JC-1-labeled mitochondria and free dye were separated by centrifugation at 14,000 × *g* for 15 min. The pellet was washed 2 times with Assay buffer, then re-suspended in 100 μl Assay buffer. The mitochondria suspensions were transferred to 96-well black wall plates. Fluorescence intensity was read at excitation at 560 nm with emission at 595 nm for red fluorescence, and at excitation at 485 nm with emission at 535 nm for green fluorescence using the Perkin Elmer VICTOR^3^ V 1420 Multilabel Counter (Wallac Oy).

For immunofluorescence microscopy, 10 μl of the JC-1 staining solution was added to each well of the 24-well plate at a density of 0.5 × 10^5^ neurons/well in 200 µl culture medium and incubated for 15 min at 37 °C in a CO_2_ incubator. After incubation, the cells were washed 2 times with Assay buffer. After washing, 200 μl of the Assay buffer was added to each well and the cells were analyzed immediately using an Olympus FluoView FV1200 Confocal Laser Scanning Microscope (Olympus America, Inc., Melville, NY) with a sequential scanning mode to minimize cross-talk among channels in multi-color images.

### Cardiolipin synthase 1 (CLS1) siRNA interference

Cultured spinal cord neurons were transfected with 50 nM cardiolipin synthase 1 (CLS1) siRNA (Santa Cruz Biotechnology) or its control siRNA using Lipofectamine® LTX and Plus™ Reagent according to the manufacturer’s instructions (Invitrogen). Briefly, Lipofectamine LTX was diluted to 4% with Opti-MEM medium and Plus reagent was added into the 1 μM CLS1 siRNA diluted with Opti-MEM medium. Then, the diluted Lipofectamine LTX and the CLS1 siRNA with Plus reagent were gently mixed (1:1 ratio) by pipetting. The mixture was incubated for 5 min at room temperature to allow for siRNA-lipid complex formation. Finally, the mixture was added to cultured spinal cord neurons for the siRNA transfection at 50 nM final concentration. With this protocol, 75% transfection efficiency was obtained in neuronal culture as measured with Cy™3 dye-labeled Pre-miR™ Negative Control #1 (17120, Invitrogen) (Supplementary Fig. 5). At 24 h after transfection, CL content was measured in isolated mitochondria using 10-N-nonyl-Acridin Orange (NAO) method. The CL was knockdown by 66.4% with the CLS1 siRNA treatment. In a subset of cultures with CLS1 siRNA, activated caspase-3/7 positive neurons were examined using CellEvent™ Caspase-3/7 Green Detection Reagent (Invitrogen) and apoptotic cells with activated caspase-3/7 showed bright green nuclei.

### Cardiolipin examination with 10-N-nonyl-Acridin Orange (NAO) method

10-N-nonyl-Acridine orange bromide (NAO, Invitrogen), which binds with high affinity to cardiolipin (CL) was used to determine CL levels as described previously [70–72] with minor modification. Briefly, mitochondria (20 µg) were incubated in standard incubation medium (125 mM KCl, 3 mM KH2PO4, 0.5 mM MgCl2, 3 mM glutamate, 3 mM succinate, 0.1% BSA, 10 mM HEPES, pH 7.4) with NAO (100 μM) at 37 °C for 15 min. NAO-labeled mitochondria and free dye were separated by centrifugation at 14,000 × *g* for 15 min. The pellet was washed with PBS, then re-suspended in the standard incubation medium. The cardiolipin-bounded NAO was measured at 535 nm after excitation at 485 nm using a Perkin Elmer VICTOR^3^ V 1420 Multilabel Counter (Wallac Oy, Turku, Finland).

### Phospho-cPLA_2_ (p-cPAL_2_) immuno-electron microscopy

Immuno-electron microscopy was performed according to our standard protocol as described previously [73]. In brief, rats were deeply anesthetized and perfused transcardially with 500 ml of 0.1 M phosphate-buffer (PB, pH 7.4) containing 0.1% glutaraldehyde, 4% formaldehyde and 15% saturated picric acid. Coronal spinal cord vibratome sections (50 µm) were preincubated in 0.1 M PB containing 25% sucrose and 10% glycerol for 1 h and then freeze-thawed with liquid nitrogen for enhancement of penetration of antibody in the immunohistochemical reaction. A pre-embedding immunohistochemical method was used for phosphorylated cPLA_2_ (p-cPLA_2_) staining in the anterior horn of spinal cord sections [73]. The sections were incubated in primary polyclonal rabbit anti-p-cPLA_2_ antibody (1:50, Cell Signaling, Boston, MA) for 24 h at room temperature (RT). After several washes in TBS, the tissue sections were incubated with biotinylated donkey anti-rabbit IgG (1:200, Millipore, CA) overnight at RT. The sections were then incubated with avidin-biotin-peroxidase complex (1:50, Elite ABC Kit; Vector Laboratories Burlingame, CA) for 6 h. The reaction product was shown by incubation for 20–30 min in 0.05 M Tris-HCl (pH 7.6) containing 0.02% (w/v) 3,3’-diaminobenzidine (DAB)-4HCl (Dojindo, Tokyo, Japan) and 0.003% (v/v) H_2_O_2_. Then, the sections were placed in 0.1 M PB containing 1% OsO_4_ for 1 h and then counterstained with 1% uranyl acetate in 70% ethanol for 1 h. After dehydration, the sections were mounted on silicon-coated glass slides and flat-embedded in epoxy resin (Durcupan; Fluka, Buchs, Switzerland). Once the resin had polymerized, small pieces containing p-cPLA_2_ or anterior horn of spinal cord were cut out from the flat-embedded sections, and selected tissue pieces were cut into 60-nm-thick sections on an ultramicrotome (Reichert-Nissei Ultracut S; Leica, Vienna, Austria). To avoid false negatives, only ultrathin sections in the first 1.5 μm from the surface of the tissue block were examined. The ultrathin sections were mounted on single-slot grids coated with piloform membrane and examined with a JEM-1400 electron microscope (JEM, Tokyo, Japan) and the digital micrographs were captured by VELETA (Olympus,Tokyo, Japan).

### Behavioral assessments

All behavioral tests were blindly performed. The Basso, Beattie, and Bresnahan (BBB) locomotor test was performed, starting 3 days post-SCI, weekly up to 6 weeks post-SCI according to a method published previously [68]. During the evaluation, animals were allowed to walk freely on the open-field surface for 4 minutes while being observed by two scorers lacking knowledge of the experimental groups. The BBB scale (0–21) represents sequential recovery stages and categorizes combinations of rat joint movement, hindlimb movements, stepping, forelimb and hindlimb coordination, trunk position and stability, paw placement, and tail position.

Grid walking was used also to assess hindlimb locomotor deficits [59]. Footfalls were evaluated at 3 and 5 weeks post-SCI. During the test, the rats were allowed to walk on a plastic mesh (3 × 3 square feet) containing 4.5 × 5 cm diamond holes. Total hindlimb footfalls were counted by two observers unaware of the experimental groups during each trial. For testing, each animal was placed on the grid and allowed to freely move until at least 100 steps have been taken by the hindlimbs. During this period, the number of steps and footfalls (fall of the hindlimb, including at least the ankle joint, through the grid surface) was counted individually for each hindlimb. A ratio between foot faults and total steps was calculated.

Footprint analysis was used to examine the stepping patterns of the rats according to previously published [74] with modification. The animals’ hind paws were inked with blue dye, and the animals were required to traverse a narrow plexiglass trough (7.5 cm wide by 115 cm long) lined with white paper. Three separate traverses of the track (trials) were recorded per testing session. A minimum of 5 consecutive footprints were assessed to determine values for the trial, and the 3 trials were averaged to obtain the values for each parameter assessed per session. Six parameters including toe spread, paw length, paw rotation, stride length, stride width, and intermediary toes were analyzed.

Bladder function was also assessed. At 2 weeks after SCI, animals were placed in a metabolic cage (Braintree Scientific, Braintree, MA) for 16 h with ample water and food during the period of urine collection. After 16 h, residual urine was collected by expressing the bladder. A ratio between residual urine and total urine was calculated for bladder function.

### Histological assessments

After 6 weeks of behavioral evaluation, all of the sham-operated rats (*n* = 4) and SCI rats (*n* = 10/group) that had received different treatments were perfused for histologic analysis. Spinal cord segments (15 mm) containing the epicenter were isolated from each animal, embedded, and cut into 25 μm-thick serial sections (250 μm apart and spanning the entire rostro caudal extent of the lesion). One set of the sections was stained for myelin with Luxol fast blue, and the other was counterstained with cresyl violet–eosin. The lesion and spared white matter area of the injured cord were visualized, outlined, and quantified using an Olympus BX60 microscope equipped with a Neurolucida system (MicroBrightField, Colchester, VT). An unbiased estimation of the percentage of spared tissue and lesion volume were calculated using the Cavalieri method [59].

### Statistical analysis

All statistical analyses were performed using GraphPad Prism software (version 7.00, La Jolla California USA). All data are presented as mean ± s.e.m. values and were analyzed by Student’s *t*-tests or analyses of variance (ANOVA; one-way or two-way as appropriate) followed by *post hoc* Dunnett or Tukey’s multiple comparison test. A *P* value of <0.05 was considered statistically significant.

## Supplementary information

Supplementary Figures

All Western blot images

Check list

## Data Availability

All data needed to evaluate the conclusions in the paper are present in the paper. Additional data related to this paper may be requested from the corresponding author.

## References

[1] Spinal Cord Injury (SCI (2016). 2016 facts and figures at a glance. J Spinal Cord Med.

[2] Rabchevsky AG, Patel SP, Springer JE (2011). Pharmacological interventions for spinal cord injury: where do we stand? How might we step forward?. Pharm Ther.

[3] Liu NK, Titsworth WL, Xu XM. Phospholipase A2 in CNS disorders: implication on traumatic spinal cord and brain injuries. In: Lajtha A (ed). *Handbook of Neurochemistry and Molecular Neurobiology*. Springer: New York, 2009, pp 321–41.

[4] Farooqui AA, Yang H-C, Rosenberger TA, Horrocks LA (1997). Phospholipase A2 and its role in brain tissue. J Neurochem.

[5] Farooqui AA, Ong WY, Horrocks LA (2004). Biochemical aspects of neurodegeneration in human brain: involvement of neural membrane phospholipids and phospholipases A2. Neurochem Res.

[6] Wymann MP, Schneiter R (2008). Lipid signalling in disease. Nat Rev Mol Cell Biol.

[7] Hall ED, Wang JA, Bosken JM, Singh IN (2016). Lipid peroxidation in brain or spinal cord mitochondria after injury. J Bioenerg Biomembr.

[8] Kagan VE, Chu CT, Tyurina YY, Cheikhi A, Bayir H (2014). Cardiolipin asymmetry, oxidation and signaling. Chem Phys Lipids.

[9] Liu NK, Xu XM (2010). Phospholipase A2 and its molecular mechanism after spinal cord injury. Mol Neurobiol.

[10] Hall ED, Braughler JM (1986). Role of lipid peroxidation in post-traumatic spinal cord degeneration: a review. Cent Nerv Syst Trauma.

[11] Braughler JM, Hall ED (1992). Involvement of lipid peroxidation in CNS injury. J Neurotrauma.

[12] Braughler JM, Hall ED (1989). Central nervous system trauma and stroke. I. Biochemical considerations for oxygen radical formation and lipid peroxidation. Free Radic Biol Med.

[13] Chicco AJ, Sparagna GC (2007). Role of cardiolipin alterations in mitochondrial dysfunction and disease. Am J Physiol Cell Physiol.

[14] Gonzalvez F, Gottlieb E (2007). Cardiolipin: setting the beat of apoptosis. Apoptosis.

[15] Kiebish MA, Han X, Cheng H, Chuang JH, Seyfried TN (2008). Cardiolipin and electron transport chain abnormalities in mouse brain tumor mitochondria: lipidomic evidence supporting the Warburg theory of cancer. J Lipid Res.

[16] Kagan VE, Tyurin VA, Jiang J, Tyurina YY, Ritov VB, Amoscato AA (2005). Cytochrome c acts as a cardiolipin oxygenase required for release of proapoptotic factors. Nat Chem Biol.

[17] Bayir H, Tyurin VA, Tyurina YY, Viner R, Ritov V, Amoscato AA (2007). Selective early cardiolipin peroxidation after traumatic brain injury: an oxidative lipidomics analysis. Ann Neurol.

[18] Ji J, Kline AE, Amoscato A, Samhan-Arias AK, Sparvero LJ, Tyurin VA (2012). Lipidomics identifies cardiolipin oxidation as a mitochondrial target for redox therapy of brain injury. Nat Neurosci.

[19] Han X, Yang J, Yang K, Zhao Z, Abendschein DR, Gross RW (2007). Alterations in myocardial cardiolipin content and composition occur at the very earliest stages of diabetes: a shotgun lipidomics study. Biochemistry.

[20] Basova LV, Kurnikov IV, Wang L, Ritov VB, Belikova NA, Vlasova II (2007). Cardiolipin switch in mitochondria: shutting off the reduction of cytochrome c and turning on the peroxidase activity. Biochemistry.

[21] Raemy E, Martinou JC (2014). Involvement of cardiolipin in tBID-induced activation of BAX during apoptosis. Chem Phys Lipids.

[22] Han X, Yang K, Yang J, Cheng H, Gross RW (2006). Shotgun lipidomics of cardiolipin molecular species in lipid extracts of biological samples. J Lipid Res.

[23] Krainz T, Gaschler MM, Lim C, Sacher JR, Stockwell BR, Wipf P (2016). A mitochondrial-targeted nitroxide is a potent inhibitor of ferroptosis. ACS Cent Sci.

[24] Ma YH, Zeng X, Zhang K, Zeng YS (2012). A new in vitro injury model of mouse neurons induced by mechanical scratching. Neurosci Lett.

[25] Hsu YH, Dumlao DS, Cao J, Dennis EA (2013). Assessing phospholipase A2 activity toward cardiolipin by mass spectrometry. PLoS One.

[26] Buckland AG, Kinkaid AR, Wilton DC (1998). Cardiolipin hydrolysis by human phospholipases A2. The multiple enzymatic activities of human cytosolic phospholipase A2. Biochim Biophys Acta.

[27] Pope S, Land JM, Heales SJ (2008). Oxidative stress and mitochondrial dysfunction in neurodegeneration; cardiolipin a critical target?. Biochim Biophys Acta.

[28] Liu NK, Deng LX, Zhang YP, Lu QB, Wang XF, Hu JG (2014). Cytosolic phospholipase A2 protein as a novel therapeutic target for spinal cord injury. Ann Neurol.

[29] Zhao Z, Liu N, Huang J, Lu PH, Xu XM (2011). Inhibition of cPLA2 activation by Ginkgo biloba extract protects spinal cord neurons from glutamate excitotoxicity and oxidative stress-induced cell death. J Neurochem.

[30] Chan RB, Di Paolo G (2012). Knockout punch: cardiolipin oxidation in trauma. Nat Neurosci.

[31] Hoye AT, Davoren JE, Wipf P, Fink MP, Kagan VE (2008). Targeting mitochondria. Acc Chem Res.

[32] Wipf P, Xiao J, Jiang J, Belikova NA, Tyurin VA, Fink MP (2005). Mitochondrial targeting of selective electron scavengers: synthesis and biological analysis of hemigramicidin-TEMPO conjugates. J Am Chem Soc.

[33] Ji J, Baart S, Vikulina AS, Clark RS, Anthonymuthu TS, Tyurin VA (2015). Deciphering of mitochondrial cardiolipin oxidative signaling in cerebral ischemia-reperfusion. J Cereb Blood Flow Metab.

[34] Khan T, Havey RM, Sayers ST, Patwardhan A, King WW (1999). Animal models of spinal cord contusion injuries. Lab Anim Sci.

[35] Widenfalk J, Lundstromer K, Jubran M, Brene S, Olson L (2001). Neurotrophic factors and receptors in the immature and adult spinal cord after mechanical injury or kainic acid. J Neurosci.

[36] Lasiene J, Shupe L, Perlmutter S, Horner P (2008). No evidence for chronic demyelination in spared axons after spinal cord injury in a mouse. J Neurosci.

[37] Baker KA, Hagg T (2005). An adult rat spinal cord contusion model of sensory axon degeneration: the estrus cycle or a preconditioning lesion do not affect outcome. J Neurotrauma.

[38] Walker CL, Fry CME, Wang J, Du X, Zuzzio K, Liu NK (2019). Functional and histological gender comparison of age-matched rats after moderate thoracic contusive spinal cord injury. J Neurotrauma.

[39] Basso DM, Beattie MS, Bresnahan JC (1996). Graded histological and locomotor outcomes after spinal cord contusion using the NYU weight-drop device versus transection. Exp Neurol.

[40] Luchetti S, Beck KD, Galvan MD, Silva R, Cummings BJ, Anderson AJ (2010). Comparison of immunopathology and locomotor recovery in C57BL/6, BUB/BnJ, and NOD-SCID mice after contusion spinal cord injury. J Neurotrauma.

[41] Paradies G, Petrosillo G, Paradies V, Ruggiero FM (2011). Mitochondrial dysfunction in brain aging: role of oxidative stress and cardiolipin. Neurochem Int.

[42] Osman C, Voelker DR, Langer T (2011). Making heads or tails of phospholipids in mitochondria. J Cell Biol.

[43] Liu NK, Xu XM (2012). Neuroprotection and its molecular mechanism following spinal cord injury. Neural Reg Res.

[44] Tyurina YY, Poloyac SM, Tyurin VA, Kapralov AA, Jiang J, Anthonymuthu TS (2014). A mitochondrial pathway for biosynthesis of lipid mediators. Nat Chem.

[45] Moon SH, Jenkins CM, Liu X, Guan S, Mancuso DJ, Gross RW (2012). Activation of mitochondrial calcium-independent phospholipase A2gamma (iPLA2gamma) by divalent cations mediating arachidonate release and production of downstream eicosanoids. J Biol Chem.

[46] Zachman DK, Chicco AJ, McCune SA, Murphy RC, Moore RL, Sparagna GC (2010). The role of calcium-independent phospholipase A2 in cardiolipin remodeling in the spontaneously hypertensive heart failure rat heart. J Lipid Res.

[47] Malhotra A, Edelman-Novemsky I, Xu Y, Plesken H, Ma J, Schlame M (2009). Role of calcium-independent phospholipase A2 in the pathogenesis of Barth syndrome. Proc Natl Acad Sci USA.

[48] Ye C, Shen Z, Greenberg ML (2016). Cardiolipin remodeling: a regulatory hub for modulating cardiolipin metabolism and function. J Bioenerg Biomembr.

[49] Mancuso DJ, Sims HF, Han X, Jenkins CM, Guan SP, Yang K (2007). Genetic ablation of calcium-independent phospholipase A2gamma leads to alterations in mitochondrial lipid metabolism and function resulting in a deficient mitochondrial bioenergetic phenotype. J Biol Chem.

[50] Clark JD, Schievella AR, Nalefski EA, Lin L-L (1995). Cytosolic phospholipase A2. J Lipid Mediat Cell Sign.

[51] Lesnefsky EJ, Chen Q, Moghaddas S, Hassan MO, Tandler B, Hoppel CL (2004). Blockade of electron transport during ischemia protects cardiac mitochondria. J Biol Chem.

[52] Petrosillo G, Di Venosa N, Pistolese M, Casanova G, Tiravanti E, Colantuono G (2006). Protective effect of melatonin against mitochondrial dysfunction associated with cardiac ischemia- reperfusion: role of cardiolipin. FASEB J.

[53] Petrosillo G, Di Venosa N, Ruggiero FM, Pistolese M, D’Agostino D, Tiravanti E (2005). Mitochondrial dysfunction associated with cardiac ischemia/reperfusion can be attenuated by oxygen tension control. Role of oxygen-free radicals and cardiolipin. Biochim Biophys Acta.

[54] Lagerwall K, Madhu B, Daneryd P, Schersten T, Soussi B (1997). Purine nucleotides and phospholipids in ischemic and reperfused rat skeletal muscle: effect of ascorbate. Am J Physiol.

[55] Crowe MJ, Bresnahan JC, Shuman SL, Masters JN, Beattie MS (1997). Apoptosis and delayed degeneration after spinal cord injury in rats and monkeys. Nat Med.

[56] Liu XZ, Xu XM, Hu R, Du C, McDonald JW, Dong HX (1997). Neuronal and glial apoptosis after traumatic spinal cord injury. J Neurosci.

[57] Beattie MS, Farooqui AA, Bresnahan JC (2000). Review of current evidence for apoptosis after spinal cord injury. J Neurotrauma.

[58] Xun Z, Rivera-Sanchez S, Ayala-Pena S, Lim J, Budworth H, Skoda EM (2012). Targeting of XJB-5-131 to mitochondria suppresses oxidative DNA damage and motor decline in a mouse model of Huntington’s disease. Cell Rep.

[59] Liu NK, Zhang YP, Han S, Pei J, Xu LY, Lu PH (2007). Annexin A1 reduces inflammatory reaction and tissue damage through inhibition of phospholipase A2 activation in adult rats following spinal cord injury. J Neuropathol Exp Neurol.

[60] Wu J, Zhao Z, Zhu X, Renn CL, Dorsey SG, Faden AI (2016). Cell cycle inhibition limits development and maintenance of neuropathic pain following spinal cord injury. Pain.

[61] Springer JE, Azbill RD, Mark RJ, Begley JG, Waeg G, Mattson MP (1997). 4-hydroxynonenal, a lipid peroxidation product, rapidly accumulates following traumatic spinal cord injury and inhibits glutamate uptake. J Neurochem.

[62] Ates O, Cayli SR, Gurses I, Turkoz Y, Tarim O, Cakir CO (2007). Comparative neuroprotective effect of sodium channel blockers after experimental spinal cord injury. J Clin Neurosci.

[63] Springer JE, Azbill RD, Knapp PE (1999). Activation of the caspase-3 apoptotic cascade in traumatic spinal cord injury. Nat Med.

[64] Cheng H, Mancuso DJ, Jiang X, Guan S, Yang J, Yang K (2008). Shotgun lipidomics reveals the temporally dependent, highly diversified cardiolipin profile in the mammalian brain: temporally coordinated postnatal diversification of cardiolipin molecular species with neuronal remodeling. Biochemistry.

[65] Matyash V, Liebisch G, Kurzchalia TV, Shevchenko A, Schwudke D (2008). Lipid extraction by methyl-tert-butyl ether for high-throughput lipidomics. J Lipid Res.

[66] Yang K, Cheng H, Gross RW, Han X (2009). Automated lipid identification and quantification by multidimensional mass spectrometry-based shotgun lipidomics. Anal Chem.

[67] Petrosillo G, Casanova G, Matera M, Ruggiero FM, Paradies G (2006). Interaction of peroxidized cardiolipin with rat-heart mitochondrial membranes: induction of permeability transition and cytochrome c release. FEBS Lett.

[68] Liu NK, Zhang YP, Titsworth WL, Jiang X, Han S, Lu PH (2006). A novel role of phospholipase A(2) in mediating spinal cord secondary injury. Ann Neurol.

[69] Wu X, Walker CL, Lu Q, Wu W, Eddelman DB, Parish JM (2017). RhoA/Rho kinase mediates neuronal death through regulating cPLA2 activation. Mol Neurobiol.

[70] Petit JM, Maftah A, Ratinaud MH, Julien R (1992). 10N-nonyl acridine orange interacts with cardiolipin and allows the quantification of this phospholipid in isolated mitochondria. Eur J Biochem.

[71] Fernandez-Gomez FJ, Gomez-Lazaro M, Pastor D, Calvo S, Aguirre N, Galindo MF (2005). Minocycline fails to protect cerebellar granular cell cultures against malonate-induced cell death. Neurobiol Dis.

[72] Sen T, Sen N, Tripathi G, Chatterjee U, Chakrabarti S (2006). Lipid peroxidation associated cardiolipin loss and membrane depolarization in rat brain mitochondria. Neurochem Int.

[73] Ge SN, Li ZH, Tang J, Ma Y, Hioki H, Zhang T (2014). Differential expression of VGLUT1 or VGLUT2 in the trigeminothalamic or trigeminocerebellar projection neurons in the rat. Brain Struct Funct.

[74] Titsworth WL, Onifer SM, Liu NK, Xu XM (2007). Focal phospholipases A2 group III injections induce cervical white matter injury and functional deficits with delayed recovery concomitant with Schwann cell remyelination. Exp Neurol.

