# Supplementary material for: Restoring mitochondrial cardiolipin homeostasis reduces cell death and promotes recovery after spinal cord injury
Source: Cell Death Dis. 2022 Dec 20;13(12):1058. doi: 10.1038/s41419-022-05369-5 (PMC9768173; doi:10.1038/s41419-022-05369-5)
Supplement: Supplementary file 1 — Supplementary Figures [file 41419_2022_5369_MOESM1_ESM.pdf]

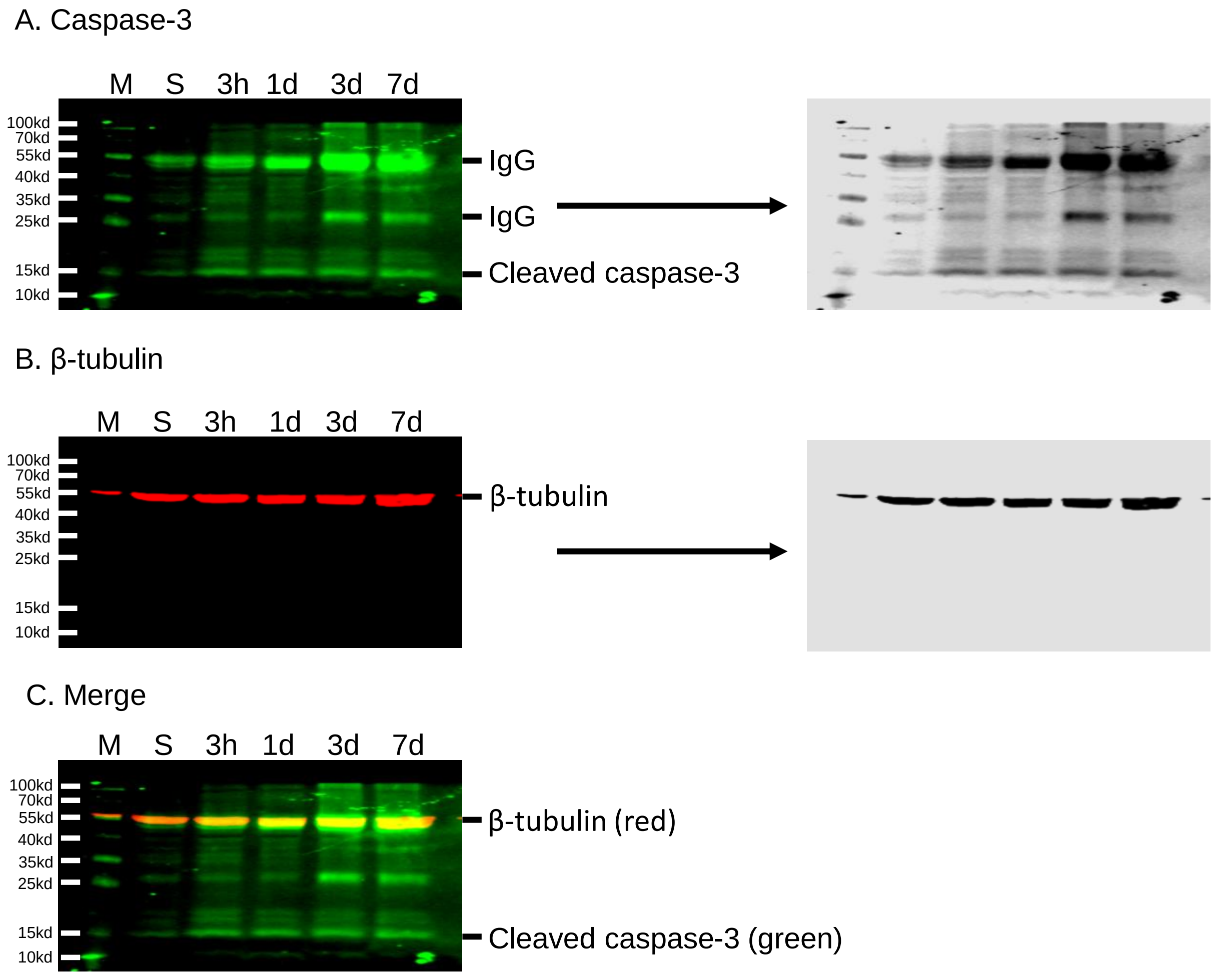

Supplementary Fig. 1. Western blot analysis of active caspase-3 expression following SCI using rabbit anti-cleaved Caspase-3 (Asp175) (5A1E) mAb (Green, Cat#: 9664, Cell Signaling Technology) and mouse anti- β-tubulin antibody as a control (red, Cat#; T5293, sigma). The representative images of active caspase-3 (A), β-tubulin (B) and their merge (C). M, Marker; S, sham.

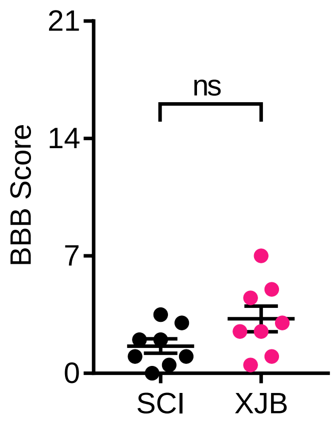

Supplementary Fig. 2. The BBB score at 3 days after SCI. ns, not significant.

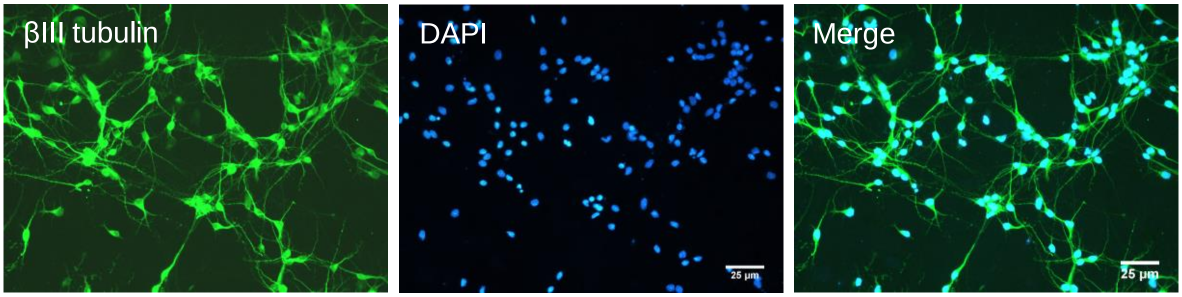

Supplementary Fig. 3. Highly purified spinal cord neurons stained with a neurite marker βIII tubulin (Green) and counterstained with a fluorescent nuclear marker DAPI (Blue). Bar = 25 µm.

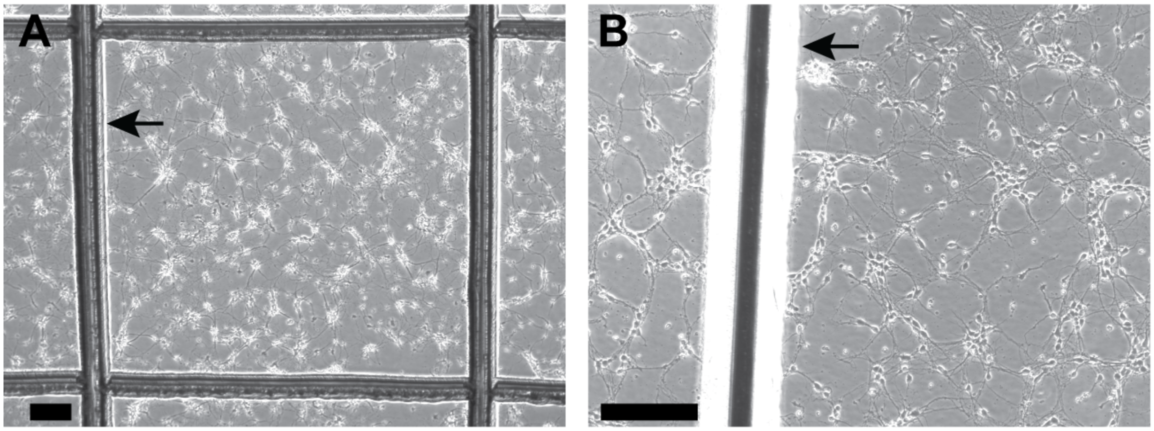

Supplementary Fig. 4. Representative images of scratch Injury of spinal cord neuronal culture. A, Low magnification; B, High magnification; Arrow, Scratch injury line. Bar = 200 µm.

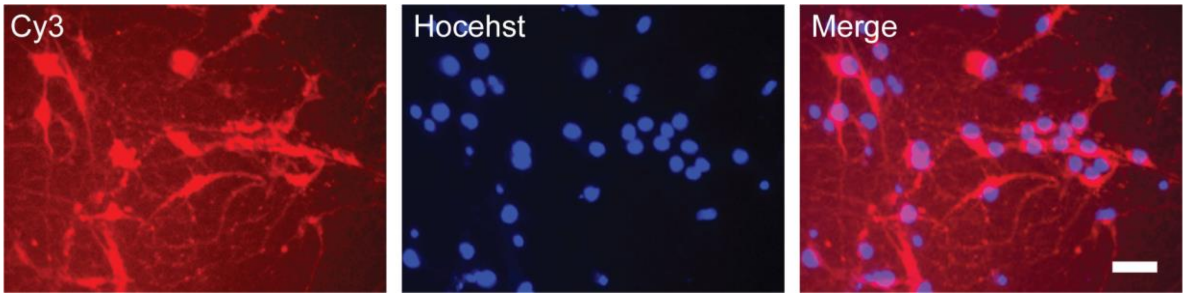

Supplementary Fig. 5. Cultured spinal cord neurons were transfected with 50 nM Cy™3 dye-labeled Pre-miR™ Negative Control #1 (Ca# 17120, Invitrogen) using Lipofectamine® LTX and Plus™ Reagent according the manufacturer's instructions (Invitrogen). The transfection efficiency was 75%. Red, Cy3 labeled neurons; Blue, nuclei. Bar = 20 µm.
