# Supplementary material for: Restoring mitochondrial cardiolipin homeostasis reduces cell death and promotes recovery after spinal cord injury
Source: Cell Death Dis. 2022 Dec 20;13(12):1058. doi: 10.1038/s41419-022-05369-5 (PMC9768173; doi:10.1038/s41419-022-05369-5)

All Western blots for Fig. 1A-C. *: representative image on the figure.

A. Cytochrome c

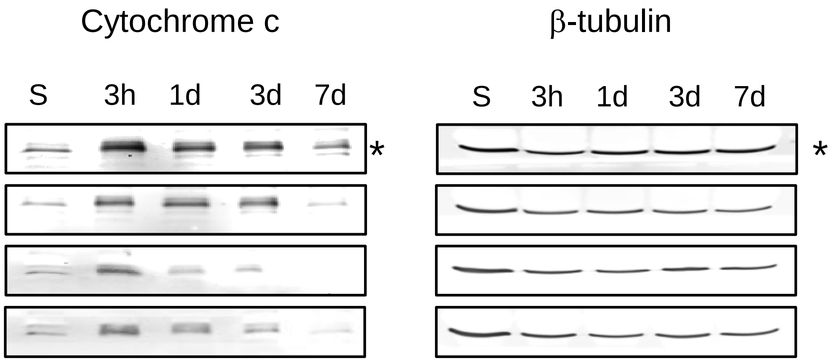

B. Smac/DIALC

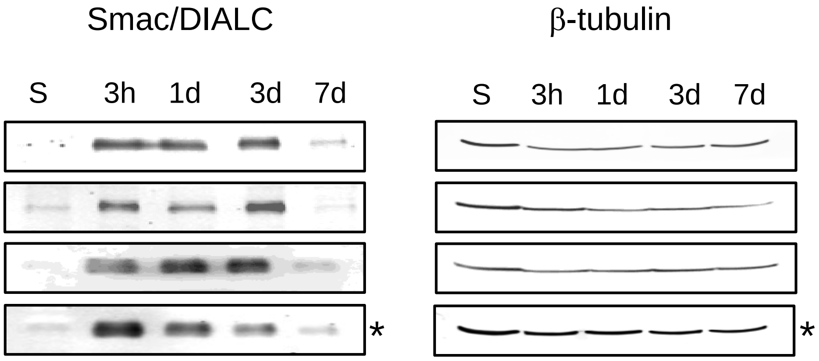

B. Active caspase-3

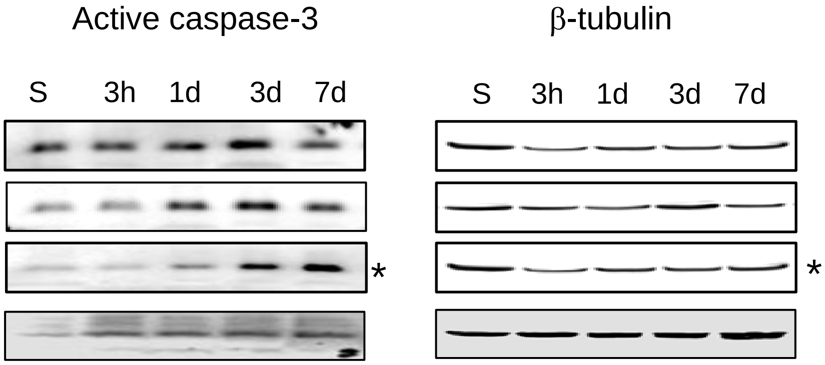

All Western blots for Fig. 4A. *: representative image on the figure.

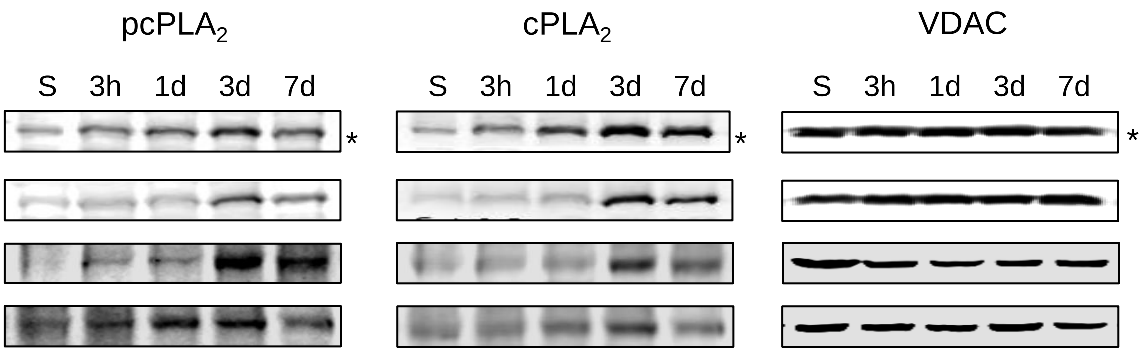

All Western blots for Fig. 4E-H. *: representative image on the figure.

Fig 4E. Cytochrome c

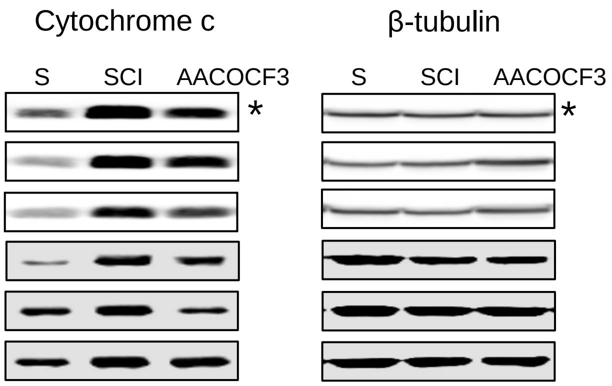

Fig 4F. Smac/DIABLO

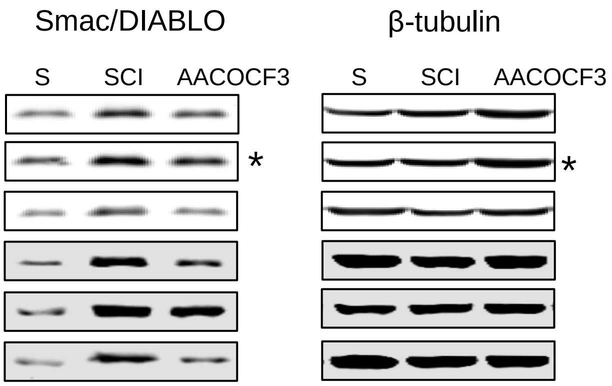

Fig 4G. Caspase-3

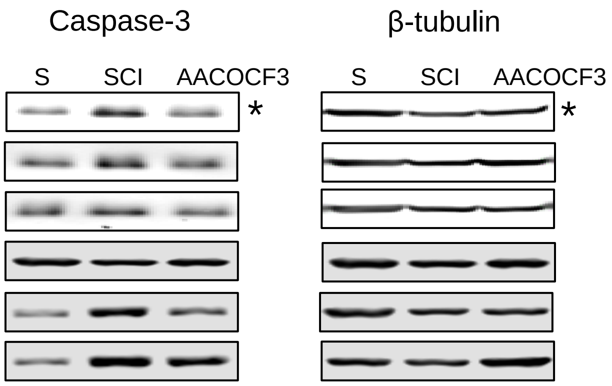

Fig 4H. PARP

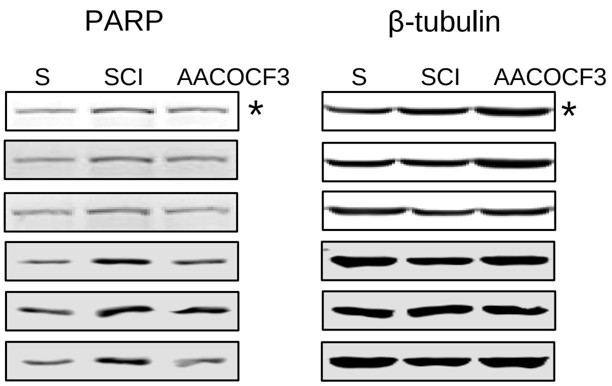

All Western blots for Fig. 4J. *: representative image on the figure.

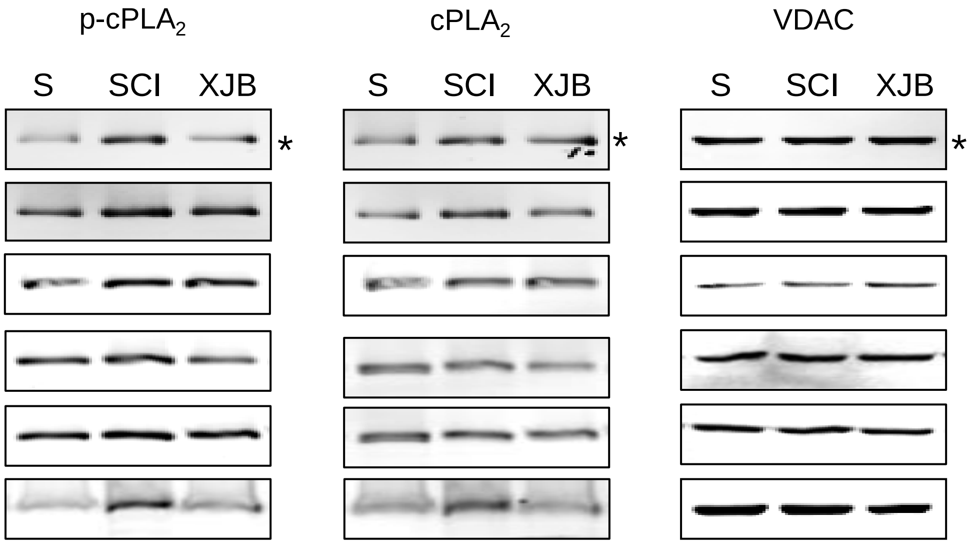

All Western blots for Fig. 5N and O. *: representative image on the figure.

N. Cytochrome c

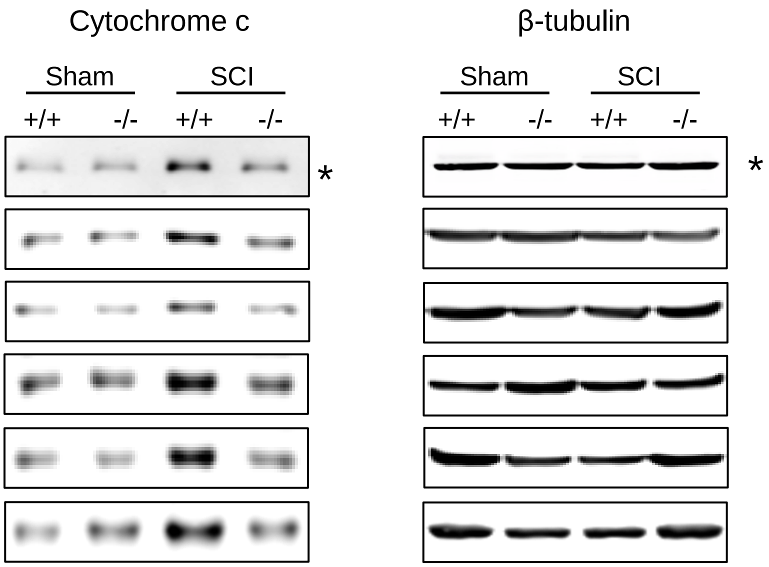

O. Active caspase-3

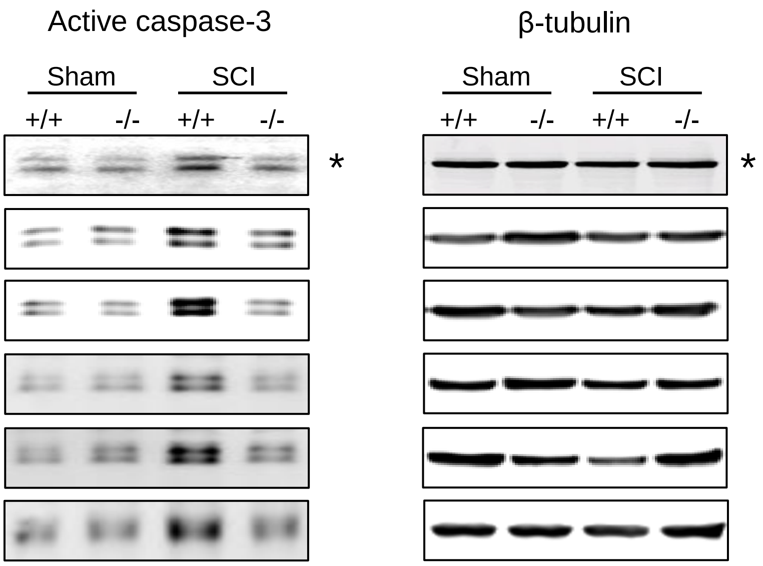

All Western blots for Fig. 6F-H. *: representative image on the figure.

Fig. 6F Cytochrome c

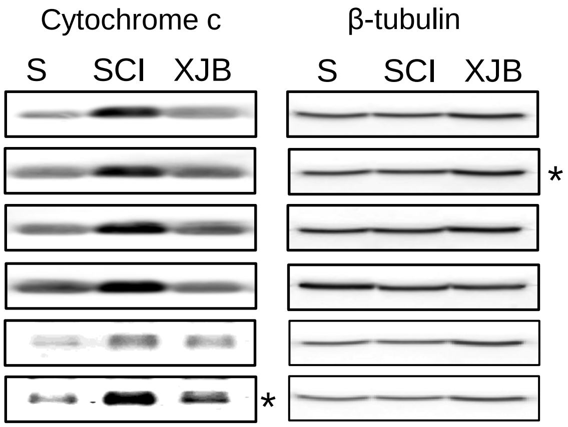

Fig. 6H Active caspase-3

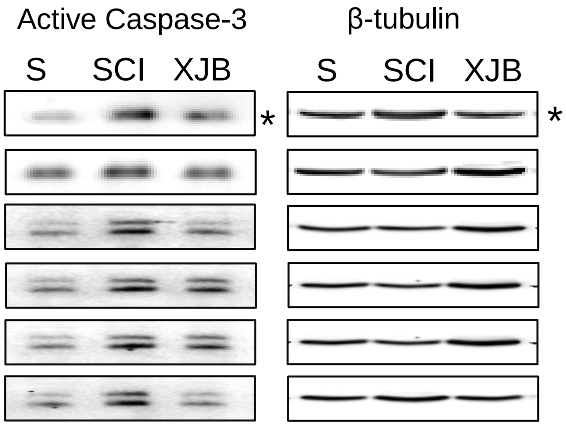

Fig. 6G Samc/DIABLO

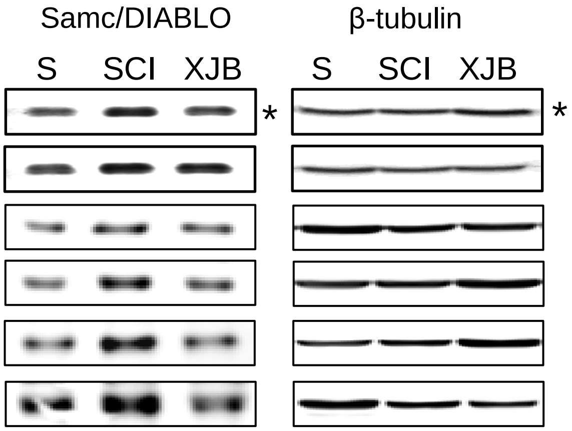

Supplement: Supplementary file 2 — All Western blot images [file 41419_2022_5369_MOESM2_ESM.pdf]
